# Highly efficient electro-oxidative synthesis of sulfoxides: synergistic effect of an optimized copper(ii) complex and covalently functionalized graphene oxide nanohybrid

**DOI:** 10.1039/d6ra06156g

**Published:** 2026-09-29

**Authors:** Elham Khodaei, Amirali Abbasi

**Affiliations:** a Department of Chemistry, Faculty of Science, Khajeh Nasir Toosi University of Technology P. O. Box 1541849611 Tehran Iran khodaeikntu@gmail.com; b Department of Chemistry Education, Farhangian University P. O. Box 14665-889 Tehran Iran

## Abstract

Integrating molecular catalysts with conductive supports is a proven strategy to enhance the kinetics of electro-catalytic processes. In this study, we developed a high-performance heterogeneous electro-catalyst, the FET-Cu–GO hybrid, specifically designed for the electro-oxidative synthesis of sulfoxides. By using TBTU as a coupling agent, we covalently anchored a copper(ii)-Schiff base complex onto a functionalized graphene oxide (GO) matrix. This approach created a stable interface that effectively prevents metal leaching and ensures rapid charge-transfer. To ensure absolute structural precision, the coordination geometry and The molecular architecture of the active site were determined by single-crystal X-ray diffraction. The synthesis and stabilization of the hybrid catalyst were further confirmed through a comprehensive set of analyses, including FT-IR, UV-vis, SEM, TEM, TGA, AAS, EDX, and powder XRD. Additionally, the electrochemical behavior and charge-transfer kinetics were analyzed *via* Cyclic Voltammetry (CV) and Chronoamperometry (CA), confirming both the stability of the active sites and the efficiency of the support. Commitment to the principles of green chemistry was central to this work; we employed hydrogen peroxide (H_2_O_2_) as a benign green oxidant and ethanol as a sustainable solvent, eliminating the need for toxic additives. When tested at an applied potential of 55 V, the system achieved 100% conversion and total selectivity in only 78 seconds. Notably, the catalyst exhibited exceptional recyclability over six consecutive cycles with no detectable metal leaching, underscored by a remarkable turnover number (TON) of 46 000. Overall, this work bridges the gap between the precision of molecular catalysts and the durability required for industrial applications, offering a promising blueprint for sustainable, high-throughput organic transformations.

## Abstract 2

A highly efficient, green electro-oxidative synthesis of sulfoxides is achieved within 78 seconds using a synergistic Cu-Schiff base and functionalized graphene oxide nanohybrid, ensuring high selectivity and recyclability.

## Graphical abstract

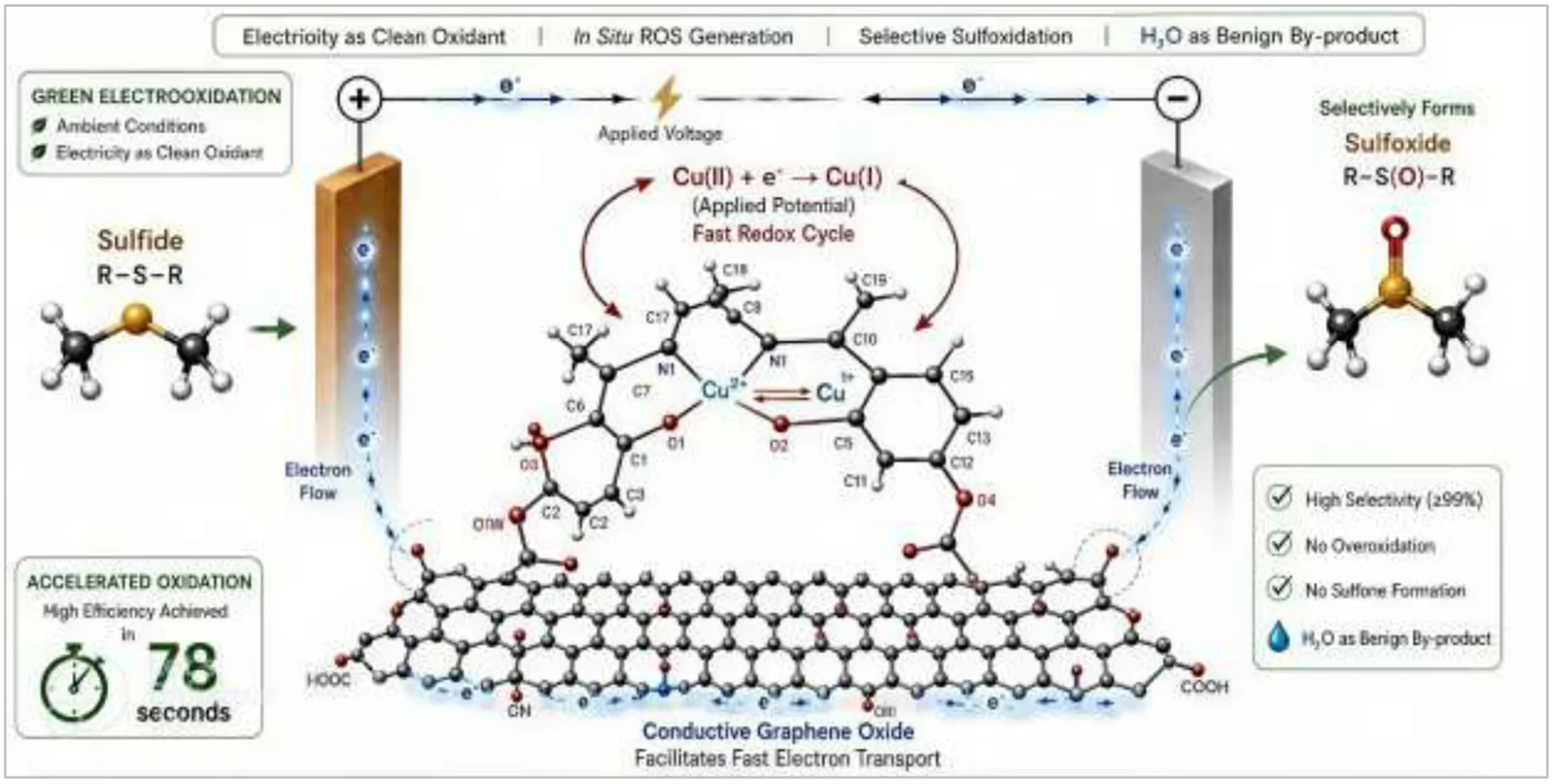

## 1 Introduction

The selective oxidation of organic sulfides to sulfoxides is essential for producing many bioactive pharmaceuticals and high-performance petrochemicals.^1^ For instance, the sulfoxide motif is key to the potency and bioavailability of proton-pump inhibitors like omeprazole and various antifungal agents.^2^ In the petrochemical industry, precise oxidation of sulfur species, including thiophenes and dibenzothiophenes, drives oxidative desulfurization to meet ultra-low sulfur diesel standards.^3,4^ Beyond this, sulfoxides are used in refining as corrosion inhibitors and for recovering precious metals from waste streams.^5^ However, producing sulfoxides on a large scale is difficult due to the trade-off between reactivity and sustainability. Many conventional routes rely on aggressive, stoichiometric oxidants and volatile solvents, which lead to waste generation and environmental issues.^6^ Heterogeneous catalysis was intended to solve catalyst recovery, but metal leaching remains a major bottleneck.^7^ When metal centers are only weakly adsorbed, they often detach into the reaction medium. This not only degrades long-term stability but also introduces metal impurities into pharmaceutical precursors, where purity standards are extremely strict.^8^ To solve this, we developed a two-dimensional charge-transfer interface using functionalized graphene oxide (GO) as a conductive, high-surface-area support. We stabilized the active copper species by covalently anchoring them to the GO matrix.^9,10^ Specifically, we used TBTU as a coupling agent to create a strong covalent bond between the copper(ii) Schiff-base complex and the GO framework.^11^ This tethering keeps the active centers spatially fixed and electronically coupled to the support, which effectively stops leaching even under harsh chemical or mechanical stress.^12^ In this work, we replaced traditional thermal activation with an external electrical driving force, moving toward a “flash-catalysis” approach.^13,14^ The interaction between the FET-Cu–GO hybrid and the applied potential triggers a rapid electron transfer (FET) mechanism that significantly speeds up the reaction. As a result, transformations that usually take hours were completed in just 78 seconds in ethanol, reaching a turnover number (TON) of 46 000. Here, we describe the synthesis, the atomic-scale structure determined by single-crystal X-ray diffraction, and the overall performance of the FET-Cu–GO-hybrid.

## 2 Experimental section

### 2.1. Materials and chemical synthesis

High-purity chemical precursors, including graphite powder, TBTU (*O*-(benzotriazol-1-yl)-*N*,*N*,*N*′,*N*′-tetramethyluronium tetrafluoroborate), and copper(ii) acetate monohydrate, were procured from Merck and Sigma-Aldrich. Absolute ethanol and the diverse range of sulfide substrates employed in this study were used as received, following stringent purity standards without further purification. Graphene oxide (GO) was synthesized from graphite powder utilizing an optimized, improved Hummers' protocol to ensure maximum oxidative exfoliation and structural consistency.

### 2.2. Physico-chemical characterization and instrumentation

The structural and morphological properties of the synthesized FET-Cu–GO-hybrid were rigorously interrogated using a comprehensive suite of advanced analytical techniques. Spectroscopic and structural analysis: the electronic transitions and optical properties were analyzed *via* UV-vis absorption spectroscopy (Unico 4802 double-beam spectrometer). The molecular architecture and purity of the ligands and organic derivatives were elucidated using ^1^H-NMR spectroscopy (300 MHz Bruker spectrometer) in DMSO-d_6_. To confirm the empirical formula and chemical composition, elemental analysis (CHN) was performed using a PerkinElmer 2400 Series II analyzer. Functional group transformations and the coordination environment were captured *via* Fourier-transform infrared (FT-IR) spectroscopy (Unicam Matson 1000) utilizing the KBr pellet technique across the 400–4000 cm^−1^ spectral range.

#### 2.2.1. X-ray diffraction studies

The precise molecular geometry and coordination environment of the copper(ii) center were elucidated *via* single-crystal X-ray diffraction (SC-XRD). Single-crystal data were collected on a Bruker Apex II diffractometer using Mo K alpha radiation (*λ* = 0.71073 angstrom). The crystal structure was solved and refined using the SHELXL program suite, with data processing performed *via* the SAINT software. To assess the long-range periodicity and phase purity of the bulk FET-Cu–GO-hybrid, powder X-ray diffraction (PXRD) was performed using a Rigaku MinX XRD diffractometer, with diffraction patterns acquired in the 2 theta range of 5–60 degrees at a scanning speed of 2 degrees per minute. This dual-XRD approach was employed to correlate the atomic-scale coordination of the active sites with the overall structural homogeneity of the conductive hybrid matrix.

#### 2.2.2. Morphological and thermal evaluation

The topographical landscape and elemental distribution were examined using field emission scanning electron microscopy coupled with energy-dispersive X-ray spectroscopy (FE-SEM and EDX, VEGA3 TESCAN) at an accelerating voltage of 20 kV. The two-dimensional morphology and internal structural integrity of the nanocomposite were further visualized through high-resolution transmission electron microscopy (TEM, Zeiss EM-1030) operating at 100 kV. Thermal stability and organic loading efficiency were quantified *via* thermogravimetric and derivative thermogravimetric analysis (TGA and DTGA, Mettler Toledo).

#### 2.2.3. Catalytic and electrochemical assessment

Metal loading and leaching kinetics were precisely monitored using atomic absorption spectroscopy (AAS, Varian AA240). The catalytic turnover and product yields were rigorously determined using gas chromatography (GC, Agilent 7890B) equipped with an SAB-5 capillary column (30 m × 0.32 mm × 0.25 micrometer) and a flame ionization detector (FID). Finally, the charge-transfer kinetics were evaluated in a standard three-electrode cell using a potentiostat–galvanostat (EG&G model 273) and a frequency response analyzer (Solartron model 1255). A glassy carbon disc and graphite were employed as the working and counter electrodes, respectively, with the reference potential calibrated against Ag/AgCl in anhydrous acetonitrile containing 0.1 M TBAH.

### 2.3. Synthesis of the Schiff base ligand (H_2_L)

We synthesized the Schiff base ligand H_2_L using a controlled condensation strategy based on previously reported protocols.^15^ For the synthesis, 5-bromo-2-hydroxybenzaldehyde (1 g, 4.97 mmol) was added to 2,2-dimethylpropylenediamine (0.254 g, 2.487 mmol) in 30 mL of absolute ethanol. The mixture was refluxed for 1 hour to complete the imine formation. After concentrating the solvent to one-quarter of its initial volume, the yellow precipitate was filtered and washed with chilled ethanol. This process afforded the target ligand with a quantitative yield of 92% (Table S1). ^1^H NMR (DMSO-d_6_, 300 MHz): *δ* 16.52–16.68 (s, 2H, OH), 9.75 (s, 1H, NH), 7.39–7.42 (m, 2H, ArH), 6.01–6.15 (m, 4H, ArH), 4.18–4.20 (m, 2H, NCH_2_), 3.33–3.65 (m, 2H, NCH–CH), 2.49–2.50 (s, 2H, CH_2_), 2.10–2.31 (s, 6H, N–CH_3_), 1.01–1.28 (m, 3H, CH_3_).

^13^C: *δ* 172.36 (CH_3_C=N), 161.67–170.81 (ArC), 103.54–130.62 (ArC), 54.06 (NCH_2_), 52.85 (NCH), 19.61 (CH_3_), 14.06 (CH_3_).

### 2.4. Preparation of the copper(ii)-Schiff base complex [CuL]

The copper(ii) complex was synthesized by adding 1 mmol (0.34 g) of the Schiff base ligand H_2_L to a boiling solution of copper(ii) nitrate trihydrate (2.1 mmol, 0.31 g) in 20 mL of absolute ethanol. The mixture was refluxed with continuous stirring for 2 hours to ensure complete coordination. The resulting precipitate was collected by filtration and purified by washing twice with chilled absolute ethanol. The product, CuL, was then dried at 80 °C. High-quality single crystals of CuC_19_H_20_N_2_O_4_ were grown by slow evaporation of the ethanolic solution at 5 °C. The complex was obtained with a high yield of 94% and showed a decomposition temperature of 265 °C (Table S2).^16^

### 2.5. Oxidative exfoliation and synthesis of graphene oxide (GO)

Graphene oxide (GO) was synthesized *via* an optimized oxidative exfoliation of high-purity graphite powder following a state-of-the-art modification of the improved Hummers' protocol.^17^ In a typical procedure, graphite (3.0 g) was meticulously dispersed into a 400 mL acidic medium of concentrated H_2_SO_4_ and H_3_PO_4_ (9 : 1 v/v ratio). Following an initial 10 min period of vigorous agitation to ensure uniform intercalation, KMnO_4_ (18.0 g) was introduced incrementally to the mixture to maintain thermal stability and prevent uncontrolled exothermic reactions. The reaction was maintained at a controlled temperature of 50 °C under continuous stirring for 24 h to facilitate the complete oxidative transformation of the graphite layers.

Upon completion, the system was equilibrated to ambient temperature and quenched through the rapid addition of 400 mL of ice-cold deionized water, followed by sustained stirring for 30 min. To ensure the quantitative removal of residual manganese species, 6 mL of 30% H_2_O_2_ was added dropwise until the characteristic color transition was observed. The resultant solid was isolated by high-speed centrifugation and subjected to an exhaustive purification sequence using repeated wash cycles with deionized water and absolute ethanol to eliminate trace metal ions, sulfates, and chlorides. The purified GO was subsequently dried under vacuum at 80 °C to yield a stable, highly exfoliated precursor.

### 2.6. Covalent engineering of the FET-Cu–GO-hybrid

The CuL complex was immobilized onto the graphene oxide (GO) framework using an ester-coupling pathway to produce the FET-Cu–GO-hybrid. In a typical procedure, 1.0 g of GO was exfoliated in anhydrous *N*,*N*-dimethylformamide (DMF) *via* ultrasonication for 30 min to create a stable colloidal suspension. To enable anchoring at the carboxylate domains, the GO framework was activated using TBTU and DIPEA. Once the activated intermediate was formed, the CuL complex was added under an inert atmosphere. The grafting process was maintained at 25 °C for 24 h to ensure sufficient coordination density across the two-dimensional lattice.

As shown in Fig. 1, the immobilization is based on a covalent linkage between the carboxylic acid groups of the GO sheets and the amine/hydroxyl functionalities of the Schiff base ligand. This tethering strategy, mediated by TBTU-induced condensation, suppresses metal leaching and enhances the long-term stability of the copper(ii) active centers. The resulting FET-Cu–GO-hybrid was isolated by centrifugation and purified through multiple wash cycles with chloroform and absolute ethanol to remove non-covalently adsorbed species. Finally, the catalyst was vacuum-dried at 60 °C.^18,19^

**Fig. 1:**
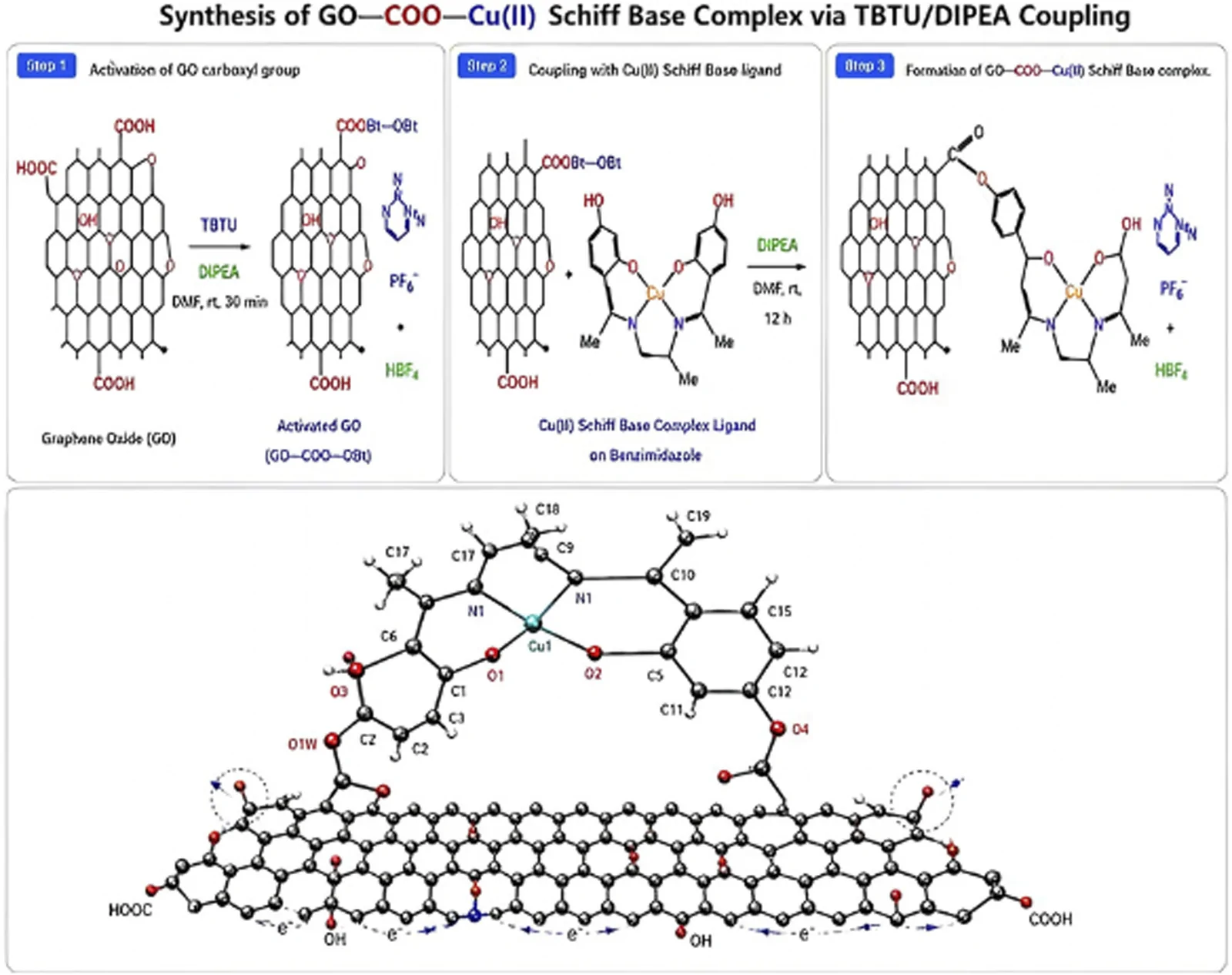
Schematic pathway for the synthesis of the FET-Cu–GO-hybrid *via* covalent attachment of the copper(ii) complex onto the graphene oxide framework.

### 2.7. Potential-driven catalytic oxidation of sulfides

The electro-catalytic performance of the FET-Cu–GO-hybrid was evaluated in a specialized electrochemical cell. In a typical procedure, 0.001 g (0.00045 mmol) of the FET-Cu–GO-hybrid was dispersed in a solution containing 150.20 mmol (21.20 mL) of methyl phenyl sulfide and 1 mL of absolute ethanol. To this mixture, 721.20 mmol (56.25 mL) of H_2_O_2_ (30% aq) was added as the oxidant, along with 33.11 mmol (0.002 g) of imidazole to modulate the coordination environment. The molar ratio of catalyst : imidazole : substrate : oxidant was maintained at 1 : 75 : 350 : 1600. The reaction was initiated by applying a constant potential of 55 V at room temperature. The transformation was completed within 78 seconds, demonstrating the high electron-transfer efficiency of the hybrid architecture. After the reaction, 1 mL aliquots were extracted and analyzed *via* Gas Chromatography (GC) to determine conversion and selectivity. This electrochemical framework was then extended to a broad scope of sulfide derivatives to demonstrate the versatility and robustness of the FET-Cu–GO-hybrid system.

### 2.8. Operational robustness and structural integrity of the FET-Cu–GO-hybrid

To evaluate the long-term operational stability and structural resilience of the FET-Cu–GO-hybrid, we investigated its catalytic endurance through successive oxidation cycles of methyl phenyl sulfide. The catalyst was recovered by centrifugation (6000 rpm, 5 min) and regenerated through ethanol washing and thermal reactivation at 80 °C to restore the active coordination sites. The FET-Cu–GO-hybrid exhibited exceptional stability, maintaining nearly quantitative turnover efficiency across sequential runs. This stability is attributed to the covalent tethering strategy, which replaces weak electrostatic adsorption with robust chemical bonding. Unlike physisorbed systems, where metal species often leach under high electric fields or solvent washing, the covalent anchoring of copper centers to the GO framework creates a high energy barrier against detachment. Additionally, the π-conjugated network of the graphene oxide acts as an electronic reservoir, modulating electron density around the copper sites and preventing the collapse of the active complex. The 2D morphology of the GO also provides a spatial confinement effect, suppressing the sintering and aggregation of active sites during high-voltage operation.

The structural robustness of the hybrid was validated through post-catalytic analyses. Atomic Absorption (AA) spectroscopy of the reaction filtrate confirmed negligible copper leaching, demonstrating that the active centers remained firmly anchored to the support. These results, along with the absence of significant alterations in the coordination environment or morphological integrity of the 2D framework, underscore the efficacy of this strategy. This sustained performance validates the FET-Cu–GO-hybrid as a durable and sustainable platform for ultrafast organic transformations, meeting the requirements for industrial-scale sustainable catalysis.^20,21^

## 3 Results and discussion

### 3.1. Characterization of the Schiff-base ligand

#### 3.1.1. IR analyses of H_2_L and CuL

The structural architecture of the synthesized Schiff base ligand was rigorously validated through FT-IR spectroscopy, providing definitive evidence of the successful nucleophilic condensation and the establishment of a highly conjugated molecular framework. The most diagnostic feature of the spectrum is the emergence of a high-intensity, sharp absorption band at 1623 cm^−1^, which is assigned to the C=N stretching vibration of the azomethine linkage. The significant bathochromic shift of this frequency relative to the precursor's carbonyl group, coupled with its pronounced intensity, confirms the quantitative conversion of the starting materials and the formation of a stable imine bond. The specific position of this peak underscores a substantial pi-electron delocalization across the aromatic system, which optimizes the electronic density of the ligand for subsequent metal coordination. Furthermore, the broad and intense absorption band centered at 3422 cm^−1^ is attributed to the O–H stretching vibrations. The pronounced broadening of this peak is a hallmark of a robust intramolecular hydrogen-bonding network, likely forming a six-membered pseudo-chelate ring between the phenolic oxygen and the imine nitrogen. This structural pre-organization is critical, as it minimizes the entropic penalty during the subsequent complexation process and ensures a high affinity toward transition metal ions.

The integrity of the aromatic scaffold is confirmed by the sharp band at 1537 cm^−1^, corresponding to the C=C skeletal vibrations of the benzene rings, and the C–H stretching bands at 2982 cm^−1^, which validate the preservation of the organic backbone. In the fingerprint region, the vibrations at 1167 cm^−1^ and 1052 cm^−1^, assigned to C–O and C–N respectively, reflect the redistribution of charge density upon the formation of the imine bond. The synergy between the π-conjugated aromatic framework and the polar imine functionality creates an electronically optimized environment, ensuring exceptional structural stability and potent chelating capabilities, consistent with previously reported data for similar Schiff base complexes (Fig. S1).^22^

#### 3.1.2. ^13^CNMR analysis

The structural fidelity of the ligand was further corroborated by ^13^C-NMR spectroscopy, which revealed a sophisticated electronic landscape. The definitive disappearance of the precursor's carbonyl resonance and the emergence of characteristic azomethine signals at 161 ppm unambiguously validate the formation of the C=N linkage. The chemical shifts in the aromatic region (103–130 ppm) reflect a highly conjugated system with significant electron density redistribution, indicative of strong intramolecular electronic communication. The precision of these chemical shifts, particularly the shielding effects observed in the phenolic substituents, aligns with quantum-chemical predictions for high-performance chelating agents. This atomic-level confirmation of the ligand's skeleton ensures that the subsequent catalytic performance is rooted in a well-defined molecular architecture (Fig. S2).^23^

#### 3.1.3. ^1^HNMR analyze

The structure and electronic environment of the synthesized Schiff base ligand were characterized by ^1^H-NMR spectroscopy, confirming the successful nucleophilic condensation. The formation of the imine linkage is evidenced by a sharp singlet at 9.75 ppm, assigned to the azomethine proton CH=N. The disappearance of the precursor's aldehyde resonance further indicates the high conversion of the starting materials. Two signals observed in the far downfield region at 16.52 and 16.67 ppm are attributed to the phenolic hydroxyl OH protons. These significant downfield shifts suggest a strong intramolecular hydrogen-bonding network, likely forming a six-membered pseudo-chelate ring between the phenolic oxygen and the imine nitrogen. This structural pre-organization reduces the entropic penalty during subsequent metal coordination, enhancing the affinity for transition metal ions. Signals in the aromatic region (6.01–7.42 ppm) exhibit splitting patterns (doublets and multiplets) consistent with the symmetric distribution of protons in the benzene rings and extended π-conjugation across the ligand. A high-intensity singlet at 2.49 ppm (integrating to 6H) confirms the presence of equivalent methyl substituents, while aliphatic resonances between 1.01 and 4.19 ppm correspond to the alkyl chain spacers. The observed chemical shifts and integration ratios confirm the purity and geometry of the ligand, consistent with the expected characteristics of high-performance chelating agents (Fig. S3).^24^

#### 3.1.4. UV-vis analysis

The electronic absorption spectrum of the Schiff base ligand exhibits a sophisticated transition profile, reflecting an extensively conjugated π-system and significant intramolecular electronic communication. The ultraviolet region is characterized by two intense, well-resolved absorption bands at approximately 280 nm, corresponding to the π to π* transitions of the aromatic phenolic rings and the azomethine C=N chromophore, respectively. The high molar absorptivity and the sharp profile of these peaks confirm the structural integrity of the conjugated framework and the efficient delocalization of electron density across the imine-linked aromatic subunits, which is essential for stabilizing the molecular architecture and facilitating long-range electronic interactions. Furthermore, distinct absorption features in the near-visible region, appearing at approximately 300 nm is assigned to the n to π* transitions originating from the non-bonding electrons of the azomethine nitrogen and the phenolic oxygen lone pairs (Table S3).^25^

### 3.2. Characterization of the Cu Schiff-base complex

#### 3.2.1. Structural analysis and catalytic implications

The structural architecture of the complex was rigorously established *via* single-crystal X-ray diffraction. The compound crystallizes in the triclinic system within the centrosymmetric space group *P*1̄, exhibiting a calculated density of 1.516 Mg m^−3^. The high reliability of the structural refinement is evidenced by the low final *R*_1_ value of 0.0441 and a goodness-of-fit (*G*_o_) of 0.949, confirming the precision of the atomic coordinates and the absence of significant thermal disorder.^26^ The molecular framework discloses a mononuclear copper(ii) center embedded within a distorted tetrahedral CuN_2_O_2_ coordination sphere (Fig. 2). This geometric distortion is primarily driven by the electronic asymmetry between the donor atoms; specifically, the observed contraction in Cu–O bond lengths relative to the Cu–N bonds induces a critical structural torsion, shifting the metal center away from a perfectly planar arrangement. This conformational strain is further characterized by a dihedral angle of 35.44 degrees between the two phenyl rings, reflecting the steric repulsion and the structural constraints imposed by the propyl bridge.^27^

**Fig. 2:**
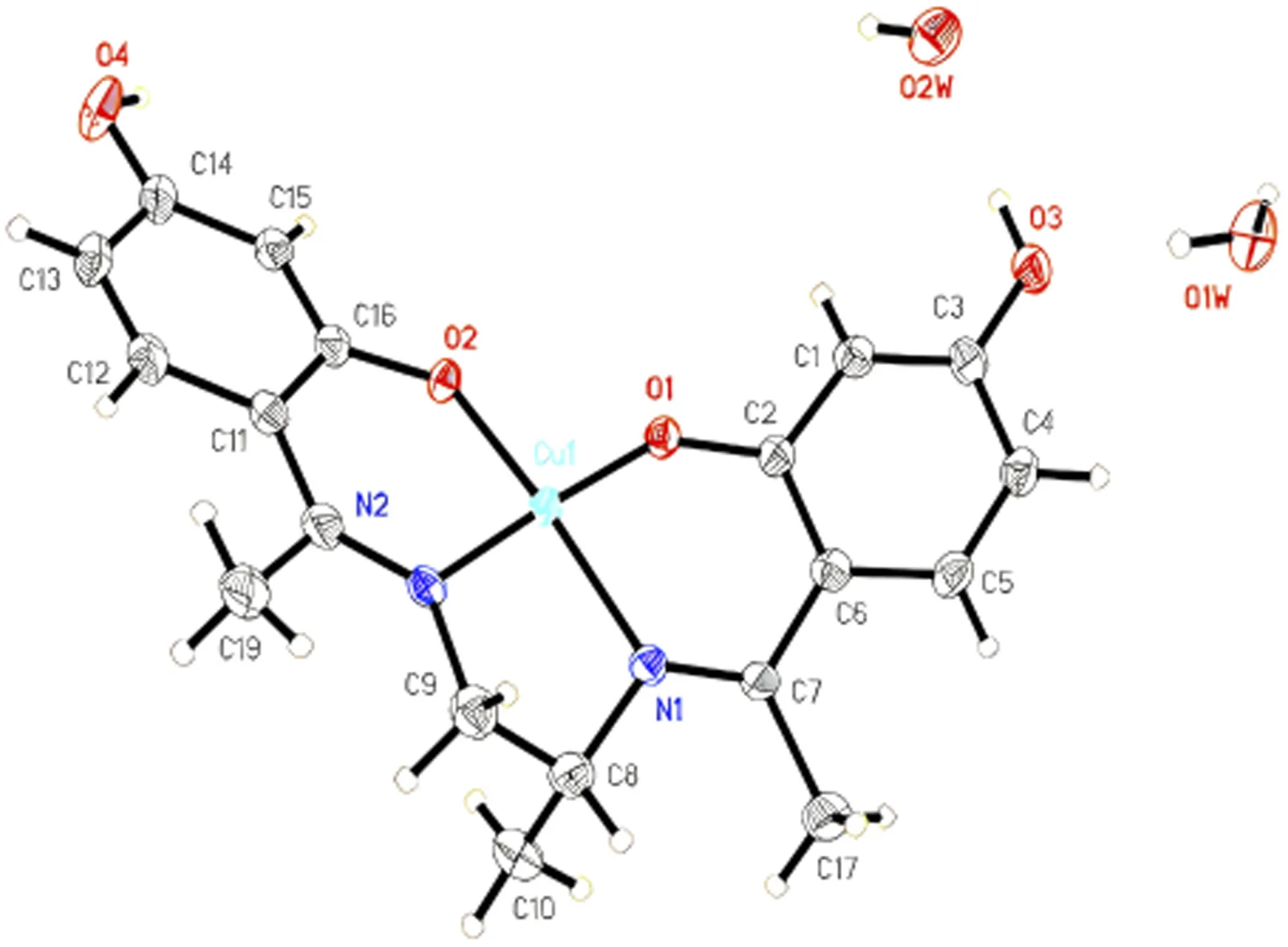
Crystal structure and ellipsoid displacement plot of CuL.

The empirical formula, C_19_H_24_CuN_2_O_6_, confirms the presence of two lattice water molecules per formula unit. These moieties, in conjunction with the pendant hydroxyl groups of the phenolic rings, facilitate a sophisticated three-dimensional supramolecular network of O–H⋯O hydrogen bonds. These interactions act as architectural anchors, stabilizing the *Z* = 2 unit cell and governing the long-range crystalline packing.^28^ Beyond its structural elegance, the coordination environment is intrinsically linked to the complex's catalytic performance. The tetradentate chelate effect, stemming from the N_2_O_2_ environment, creates a robust molecular cradle that encapsulates the Cu(ii) center. This rigid binding pocket, reinforced by the significant overlap between the metal d-orbitals and the electron-rich donor atoms, effectively suppresses metal leaching during catalytic cycles. This structural integrity ensures that the active site remains firmly anchored within the ligand framework, preventing the dissociation of the metal into the reaction medium, thereby facilitating the recovery and thereby enhancing the recyclability of the catalyst with negligible loss in efficiency (Fig. 2).^29^ The finalized structural data have been deposited with the Cambridge Crystallographic Data Centre (CCDC) under the deposition number 1537879.

Furthermore, the inherent geometric distortion of the CuN_2_O_2_ coordination sphere plays a critical role in modulating the redox properties of the metal center. The observed deviation from ideal planarity significantly reduces the reorganization energy required during the transition between oxidation states.^30^ This structural flexibility, coupled with the strong σ-donating ability of the phenolic oxygen atoms, effectively lowers the oxidation potential of the copper center. Consequently, under an applied potential, the metal center undergoes rapid oxidation, facilitating an accelerated electron transfer process. This rapid redox switching is fundamental to the overall catalytic efficiency, as it ensures the facile generation of the active oxidized species, thereby significantly enhancing the reaction kinetics of the catalyst (Table 1).^31,32^

**Table 1:** Bond lengths [Å] and angles [deg] in the structure of CuL

Bond distances		Angles	
Cu1–O2	1.8694(19)	O2–Cu1–O1	88.15(8)
Cu1–O1	1.8988(18)	O2–Cu1–N1	174.56(11)
Cu1–N1	1.933(2)	O1–Cu1–N1	91.78(9)
Cu1–N2	1.936(2)	O2–Cu1–N2	93.27(10)
O1–C1	1.320(3)	O1–Cu1–N2	177.11(10)
		N1–Cu1–N2	87.03(10)

#### 3.2.2. Coordination analysis of the Cu(ii) complex

The coordination of the copper(ii) center within the tetradentate ligand framework is unequivocally confirmed by the specific vibrational shifts observed in the FT-IR landscape. The presence of the peak at 2922 cm^−1^ identifies the characteristic stretching vibrations of the aliphatic C–H groups, confirming the structural integrity of the ligand backbone. The significant transition of the imine C=N stretching frequency toward a lower energy region is a primary diagnostic indicator of coordination, resulting from the synergistic σ-donation from the nitrogen lone pairs to the d-orbitals of the Cu(ii) ion, which effectively reduces the double-bond character of the imine linkage (Fig. S4).^33^

The involvement of the phenolic oxygen atoms in the metal-bonding process is evidenced by the strategic shift of the C–O stretching vibration, observed here at 1026.08 cm^−1^. This shift reflects the intensified covalent character of the Cu–O bond and a subsequent electronic reorganization within the aromatic rings. Furthermore, the peak at 851.41 cm^−1^ corresponds to the C–H out-of-plane deformations, while the emergence of the low-frequency mode at 158 cm^−1^ is highly indicative of the metal–ligand Cu–N/O stretching vibrations in the far-IR region. These vibrational signatures collectively define a robust N_2_O_2_ coordination environment, stabilizing the mononuclear copper(ii) center in a distorted coordination geometry.^34^

#### 3.2.3. UV-vis spectroscopic analysis of CuL complex

The high-energy absorption band observed at 235 nm is attributed to the π-to-π* transitions within the aromatic framework of the ligand, while the secondary shoulder-like feature at approximately 295 nm corresponds to the n-to-π* star transitions of the azomethine (C=N) chromophore. These high-molar extinction coefficient bands signify the successful formation of the conjugated chelate system, where the electronic density is highly delocalized across the N_2_O_2_ coordination plane (Fig. 3). A more diagnostic feature of the coordination geometry is the prominent absorption band centered at 348 nm, which is assigned to the phenolate-to-copper(ii) charge transfer (LMCT). This transition indicates a strong covalent interaction between the deprotonated phenolic oxygen atoms and the Cu(ii) d-orbitals. The broad tail extending into the visible region, with a subtle shoulder around 540–560 nm, is characteristic of the d–d electronic transitions (specifically the d_*xz*_, d_*yz*_ to d_*x*^2^−*y*^2^_ transition). The intensity and position of these bands collectively point toward a mononuclear copper(ii) center residing in a distorted square-planar or pseudo-tetrahedral geometry, where the Jahn–Teller effect further lifts the degeneracy of the d-orbitals, as corroborated by recent computational and spectroscopic models.^35^

**Fig. 3:**
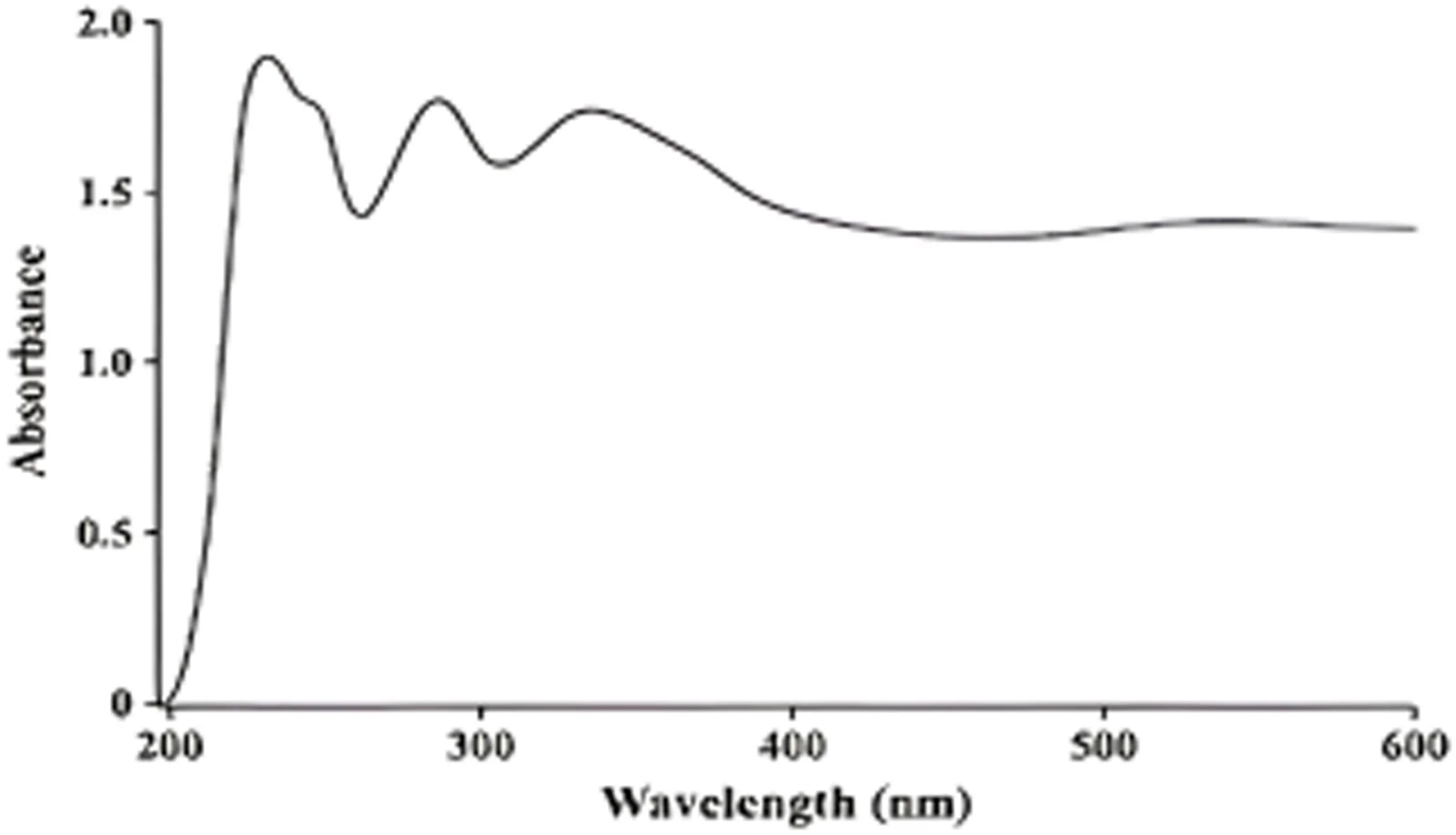
UV-vis absorption spectra of the CuL complex.

### 3.3. Characterization of the heterogeneous FET-Cu–GO-hybrid

#### 3.3.1. AAS analysis of FET-Cu–GO hybrid

To rigorously assess the efficiency of the immobilization process, the metal content of the synthesized FET-Cu–GO-hybrid was quantified *via* Atomic Absorption Spectroscopy (AAS). Utilizing a high-precision linear calibration framework, the copper loading was determined to be 1.33 mmol g^−1^. This substantial result not only corroborates the successful integration of the copper(ii) complexes onto the functionalized graphene oxide (GO) support but also signifies a strategic distribution of active centers, across the matrix. Such a loading density is indicative of an optimized stoichiometric balance, ensuring an abundance of accessible active sites while effectively preventing the agglomeration of metal centers, witch is a critical factor in maintaining the structural integrity and high catalytic activity of the system. Furthermore, the consistency between the theoretical and experimental loading underscores the robust coordinative interactions between the FET-derived complexes and the oxygen-rich moieties of the GO support, which is pivotal for enhancing the synergistic effects and long-term recyclability of the hybrid nanocatalyst.

#### 3.3.2. FT-IR analysis of FET-Cu–GO-hybrid

The chemical architecture of the CuL–GO hybrid was rigorously evaluated by comparing the FTIR signatures of pristine GO, the binary physical mixture (GO/CuL), and the chemically synthesized hybrid (Fig. 4). The spectrum of pristine GO exhibits the hallmark features of a heavily oxidized carbon lattice, with the broad –OH stretching envelope at 3412 cm^−1^ and a prominent carbonyl band at 1620 cm^−1^, reflecting the coexistence of carboxylic acids and ketones. The epoxide (C–O–C) and alkoxy (C–O) vibrations at 1222 and 1113 cm^−1^ further delineate the oxygen-rich environment of the GO surface. To establish a baseline for non-covalent interactions, the physical mixture was scrutinized. The resulting spectrum demonstrates a stoichiometric superposition of the individual components, where all characteristic vibrational frequencies are preserved without any significant shift in wavenumbers. This lack of spectral perturbation indicates that the chemical environment of the functional groups remains unperturbed under simple mechanical blending. The perceived attenuation of peak intensities is merely a dilution phenomenon and does not suggest any chemical transformation. Critically, the total absence of any absorption features in the ester carbonyl region (1700–1730 cm^−1^) in the physical mixture serves as a negative control, proving that the proximity of the CuL complex to the GO surface is insufficient to trigger the esterification process without specific synthetic conditions. In stark contrast, the CuL–GO hybrid spectrum undergoes a profound evolution, signifying a fundamental change in the molecular bonding. The most diagnostic evidence is the emergence of a sharp, high-intensity absorption band at 1721 cm^−1^ (with a subtle shoulder at 1724 cm^−1^), unambiguously attributed to the stretching vibration of an ester carbonyl (C=O). This shift from the original 1620 cm^−1^ (carboxyl) to 1721 cm^−1^ (ester) is indicative of the conversion of surface carboxylates into ester linkages, as the ester carbonyl typically vibrates at a higher frequency due to the alteration of the electronic environment and the loss of the carboxylic hydrogen-bonding network. This transition is further corroborated in the fingerprint region, where the distinct epoxide and alkoxy modes of GO (1222 and 1113 cm^−1^) and the ligand signal (1168 cm^−1^) coalesce into a broad, intensified absorption envelope, reflecting the formation of a complex C–O–C ester network. Simultaneously, the bathochromic shift of the –OH band to 3416 cm^−1^ and its accompanying decrease in intensity provide a direct spectroscopic measure of the consumption of surface hydroxyl and carboxyl groups during the condensation reaction. The cumulative evidence specifically the emergence of the ester carbonyl signal exclusively in the hybrid confirms that the CuL complex is chemically anchored to the GO framework *via* robust covalent ester bonds (Fig. 4).^35,36^

**Fig. 4:**
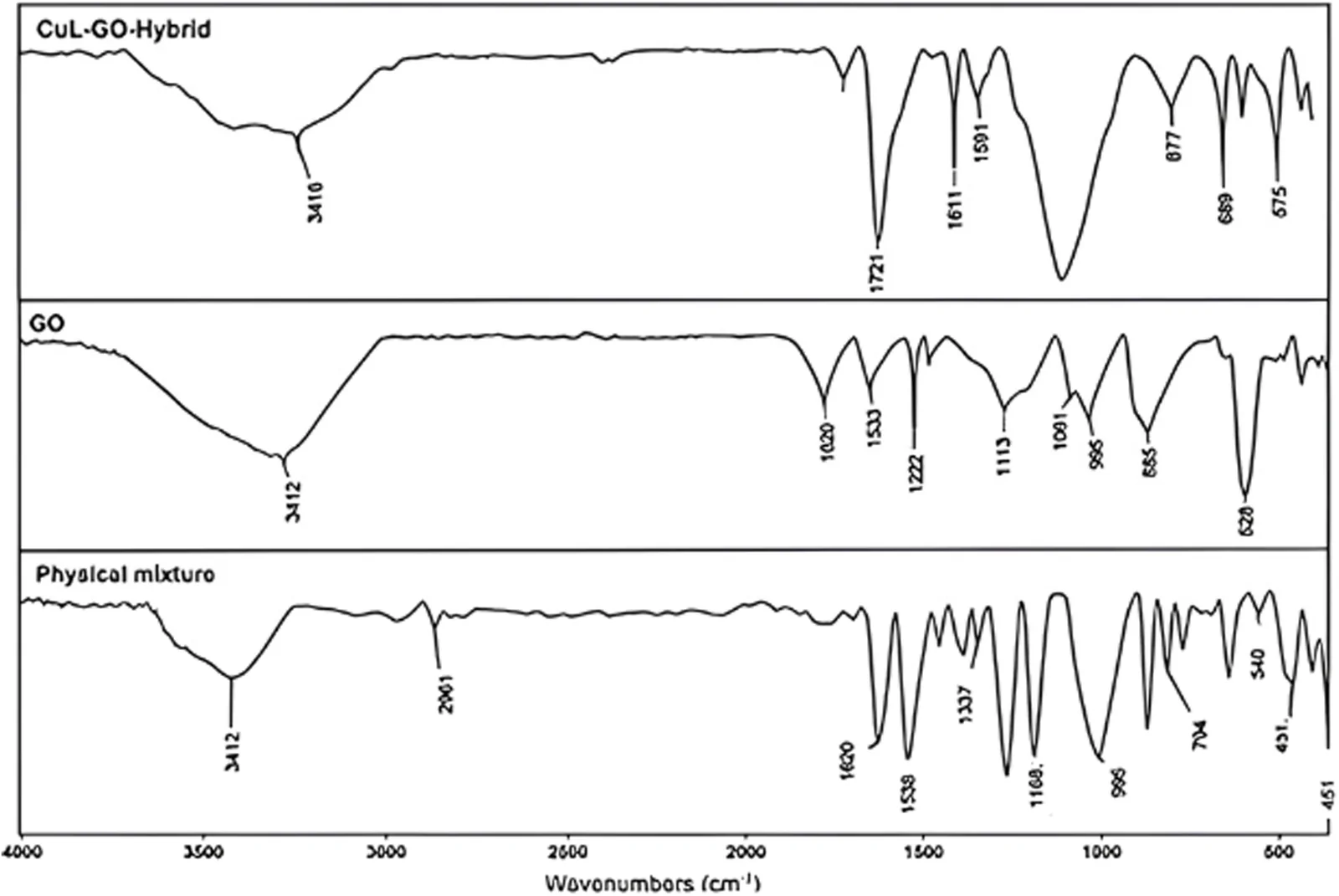
The relevant FT-IR data for GO and the FET-Cu–GO-hybrid and physical mixture.

#### 3.3.3. Morphological characterization of the FET-Cu–GO

The morphology of the FET-Cu–GO-hybrid catalyst was analyzed *via* FE-SEM, revealing a structure composed of extended, quasi-planar, and highly exfoliated sheets. In this architecture, the Cu-Schiff base complexes act as molecular spacers, effectively disrupting van der Waals attractions and preventing the re-stacking of the sheets. High-resolution imaging at 90.0k× magnification shows a homogeneous surface texture, devoid of discrete metallic clusters or nanoparticle aggregates, confirming the site-isolated, monomolecular dispersion of copper species throughout the framework. This optimized morphology increases the accessible surface area and the exposure of active Cu(ii) centers to the reactants, providing a structural basis for enhanced catalytic activity and long-term stability under reaction conditions (Fig. 5).^37^

**Fig. 5:**
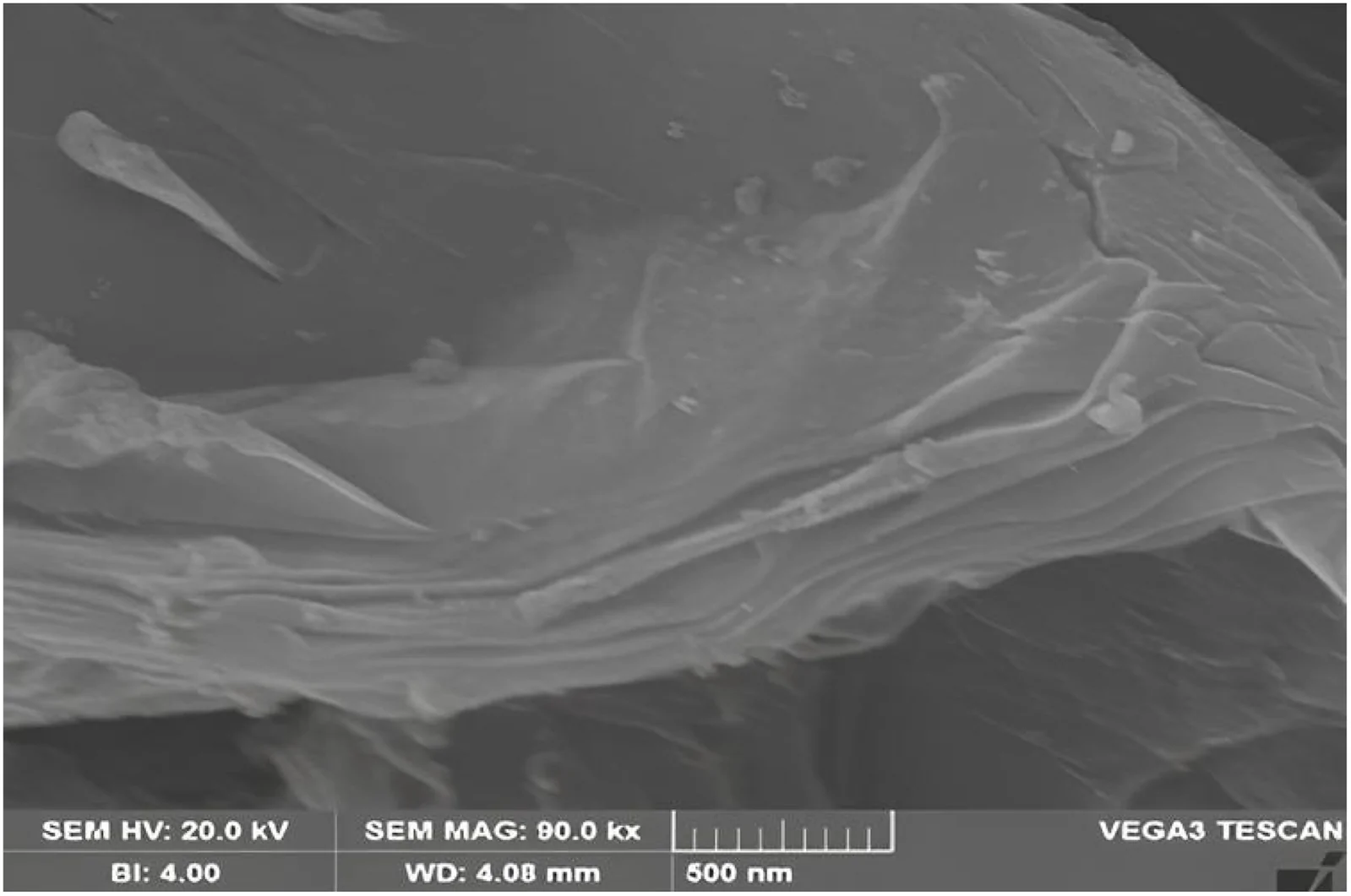
SEM images of the synthesized FET-Cu–GO-hybrid catalyst.

#### 3.3.4. Morphological analysis *via* TEM

The morphology and dispersion of the copper(ii) complex (CuL) were investigated *via* TEM to understand its interaction with the GO support (Fig. 1). For the free complex, the images show the presence of dense, massive aggregates. This morphology is primarily due to high lattice energy and strong intermolecular cohesive forces, which drive the complex to form large bulk clusters with a long-range crystalline order. In the case of the physical mixture (CuL + GO), the TEM images indicate that the bulk morphology of the complex remains largely unchanged. The CuL clusters appear as discrete aggregates merely adsorbed onto the GO surface through weak non-covalent interactions. A noticeable lack of homogeneity is observed here; the images show a ‘patchy’ distribution, with localized aggregates and large bare regions on the GO basal planes. This indicates that simple physical adsorption is insufficient to disrupt the crystalline lattice of the bulk phase or to ensure a uniform distribution across the support. In contrast, the FET-Cu–GO-hybrid shows a significant morphological transformation. The bulk aggregates are replaced by finely dispersed nanoparticles that uniformly decorate the GO sheets. These nanoparticles are highly dispersed without any observed voids, indicating that the complex is no longer in a bulk phase but has been successfully anchored as individual nanophases. This implies that the GO surface provides a stabilizing environment that suppresses crystal growth and prevents re-aggregation. In conclusion, the transition from ‘bulk aggregation’ in the free complex and ‘patchy adsorption’ in the physical mixture to the uniform nanodispersions seen in the hybrid provides a clear morphological indication of successful chemical immobilization. The formation of covalent or coordinate bonds not only prevents the aggregation of CuL but also ensures uniform coverage of the GO surface. This process breaks the long-range crystalline order of the free complex and stabilizes the active species at the nanoscale, which ultimately maximizes the accessibility of the active sites (Fig. 6).^38^

**Fig. 6:**
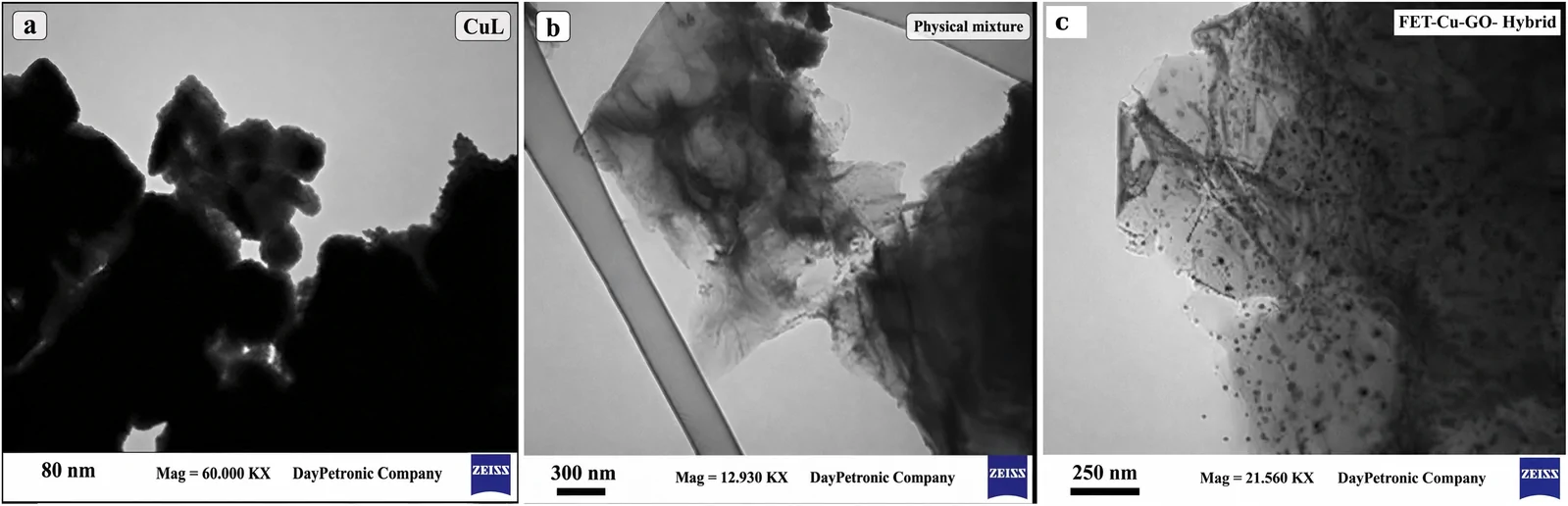
TEM analysis of the catalyst evolution: (a) the free CuL complex showing massive bulk aggregation; (b) the physical mixture (CuL + GO) exhibiting non-uniform, patchy adsorption of coarse clusters with significant vacant regions on the GO support; and (c) the FET-Cu–GO-hybrid demonstrating a complete morphological transformation into uniform nanodispersions.

#### 3.3.5. Thermal stability analysis (TGA)

The nature of the interfacial coupling between the Cu(ii) Schiff base complex (CuL) and the graphene oxide (GO) scaffold was interrogated *via* TGA/DTG analysis to determine the thermodynamic stability of the resulting architecture. To rigorously distinguish between superficial physisorption and genuine chemical hybridization, we employed a comparative framework consisting of the pristine precursors, their mechanical blend, and the engineered FET-Cu–GO hybrid. Pristine CuL exhibited a characteristic multi-step decomposition, defined by the desorption of coordinated solvent molecules (∼200 °C) and the subsequent oxidative pyrolysis of the Schiff base framework (280–450 °C), yielding a stable CuO residue. In parallel, the thermal profile of pristine GO was dominated by the lability of its oxygenated moieties, manifested as a sharp mass loss of 27.88% at ∼200 °C. Crucially, the physical mixture displayed a strictly linear additive superposition of these individual thermal signatures. The absence of any shift in onset temperatures or degradation kinetics confirms that the precursors retain their discrete electronic identities, interacting solely through non-specific van der Waals forces and π–π stacking. This additive regime provides a definitive thermodynamic baseline, ruling out any formal chemical interaction in the blend. In stark contrast, the FET-Cu–GO hybrid exhibited a profound transformation in its thermal fingerprint, signifying a transition to a synergistic regime. The attenuation of the GO deoxygenation event from 27.88% to 16.53% serves as a quantitative proxy for the consumption of oxygenated functional groups, which act as coordination anchors for the covalent tethering of CuL centers to the GO basal plane. Furthermore, the anchored ligands exhibited enhanced thermal resilience, evidenced by a broadened DTG profile and a significant shift toward higher temperatures. This stabilization suggests that the π-conjugated GO lattice functions as a protective electronic shield, modulating the coordination sphere of the Cu-complex and substantially elevating the activation energy barrier (Δ*G*) for pyrolysis.

The emergence of a unique high-temperature degradation event (600–700 °C), entirely absent in all other systems, unequivocally points to the formation of a robust, covalently-bonded organometallic interface. Collectively, the suppression of labile oxygenated species, the thermal stabilization of the ligand, and the appearance of a novel interfacial signal provide compelling evidence for the seamless chemical integration of CuL into the GO framework, yielding a structurally reinforced and thermally robust hybrid catalyst (Fig. 7).^39^

**Fig. 7:**
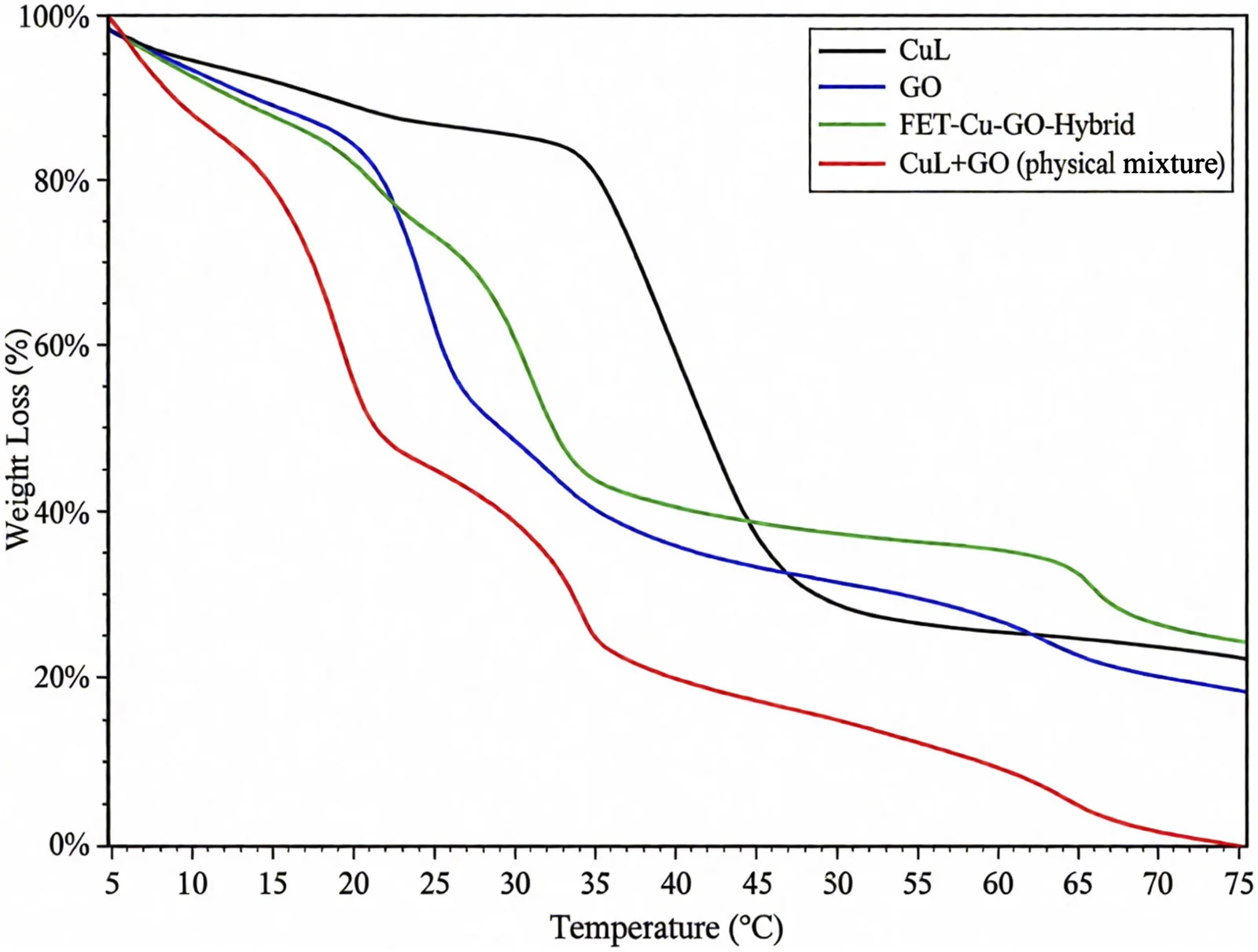
TGA thermograms of pristine CuL, pristine GO, their physical mixture, and the FET-Cu–GO hybrid.

#### 3.3.6. X-ray diffraction (XRD) analysis of FET-Cu–GO-hybrid

##### 3.3.6.1 PXRD analysis and structural characterization

The structural properties of the FET-Cu–GO-hybrid were investigated using PXRD analysis (Fig. 8). The diffraction pattern of pure GO (Fig. 8a) shows the characteristic peak at 2*θ* ≈ 9.1°, while the pure CuL complex (Fig. 8c) exhibits a series of sharp, intense reflections corresponding to its crystalline lattice. To further investigate the nature of the interaction, a physical mixture of GO and CuL was analyzed (Fig. 8d). The resulting pattern is a superposition of the two individual phases, with no detectable shifts in the 2*θ* positions, confirming that both species coexist as independent crystalline entities. The observed decrease in peak intensity in the physical mixture (d) compared to the pure complex (c) is consistent with a typical dilution effect. In contrast, the XRD pattern of the FET-Cu–GO-hybrid (Fig. 8b) indicates a significant structural change. The near-complete disappearance of the sharp reflections associated with the CuL complex, along with the broadening of the GO peak, suggests a loss of long-range crystalline order. This indicates that the CuL species are highly dispersed as a quasi-amorphous phase on the GO sheets, likely as a result of strong interfacial anchoring. The difference between the physical mixture (d) and the hybrid (b) suggests that the interaction is chemical rather than physical, leading to the disruption of the bulk crystalline lattice of the complex and the formation of an integrated hybrid architecture.^40^

**Fig. 8:**
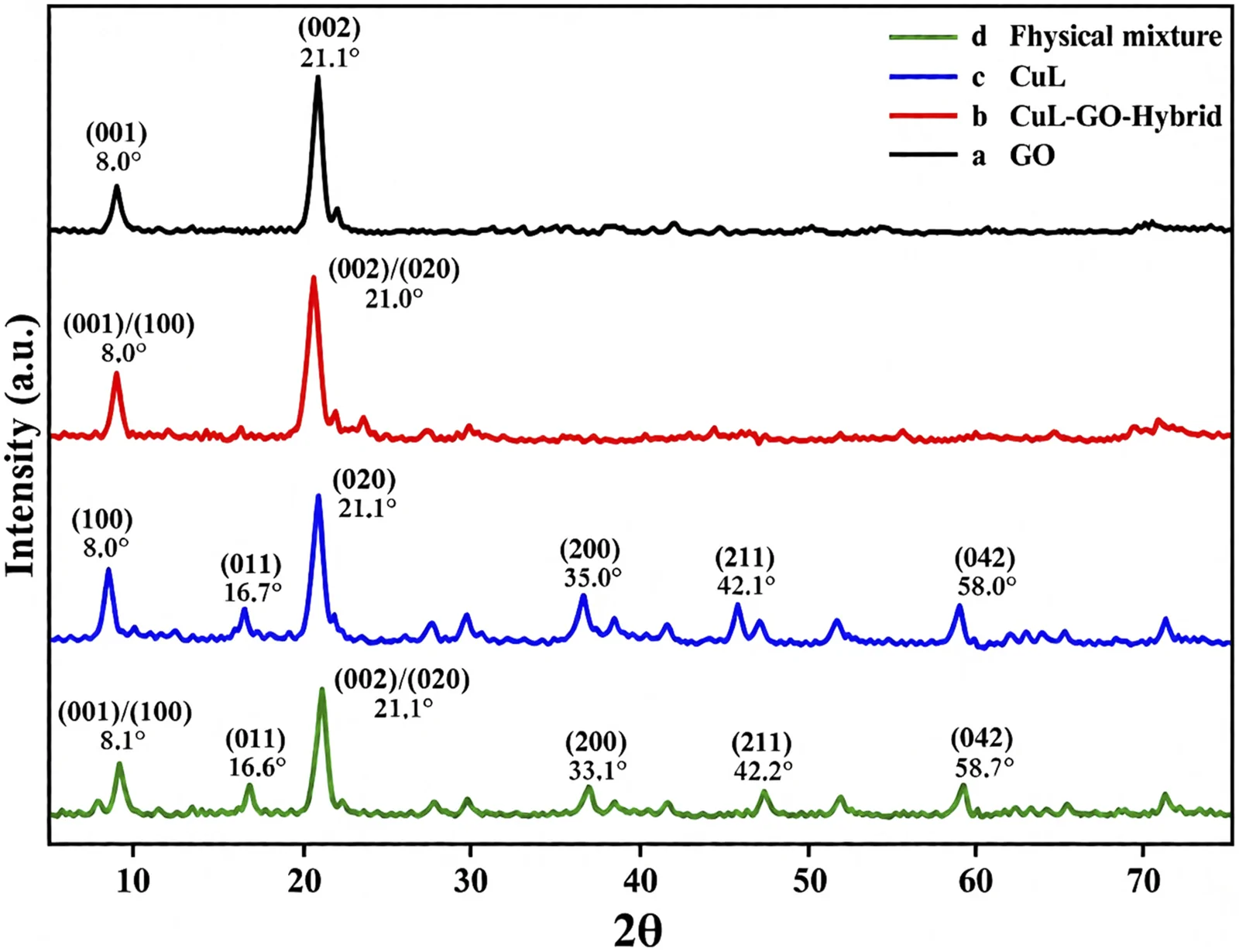
XRD patterns of the (a) GO and (b) FET-Cu–GO hybrid, (c) CuL and (d) physical mixture.

#### 3.3.7. Energy-dispersive X-ray (EDX) spectroscopy of the FET-Cu–GO-hybrid

The elemental composition and surface distribution of the synthesized hybrid catalyst were quantitatively assessed using Energy-Dispersive X-ray spectroscopy (EDX), as illustrated in the spectrum and corresponding weight percentage data. The EDX analysis confirms the presence of carbon (C), oxygen (O), copper (Cu), and sulfur (S) as the primary constituent elements. The predominant peaks for carbon (54.8 wt%) and oxygen (37.9 wt%) originate from the graphene oxide (GO) framework, representing the graphitic backbone and the residual oxygenated functional groups, respectively. Significantly, the detection of copper (5.5 wt%) provides direct evidence for the successful immobilization of the Cu(ii)-Schiff base complex onto the GO support. The presence of sulfur (1.8 wt%) further confirms the integration of the sulfur-containing ligand or auxiliary reagents used during the synthesis process. This elemental analysis validates the incorporation of the coordination complex onto the GO scaffold, establishing a robust platform for heterogeneous catalytic applications (Fig. 9).^41^

**Fig. 9:**
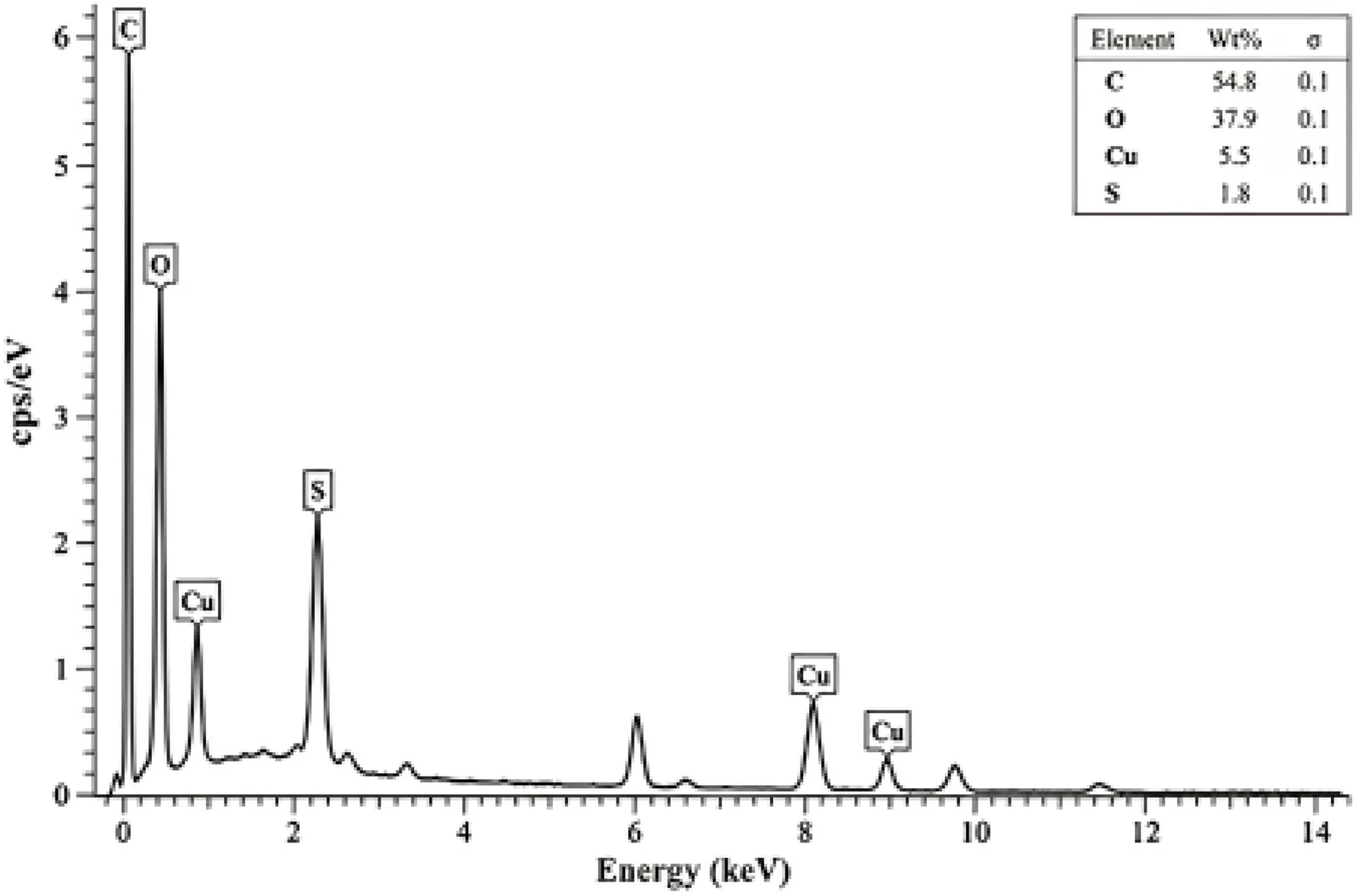
EDX spectrum of the synthesized FET-Cu–GO-hybrid.

#### 3.3.8. UV-vis spectroscopy analysis

The comparative electronic landscapes of pristine graphene oxide (GO) and the FET-Cu–GO-hybrid catalyst were scrutinized *via* UV-vis diffuse reflectance spectroscopy, as illustrated in Fig. 10. The absorption profile of GO is characterized by a high-intensity transition at approximately 230 nm, originating from the π to π* transitions of aromatic C=C bonds, and a subtle shoulder at roughly 300 nm, attributed to the n to π* star transitions of C=O moieties. Upon the integration of the Cu(ii)-Schiff base complex, the spectral features undergo a profound transformation, serving as a diagnostic fingerprint for successful immobilization.

**Fig. 10:**
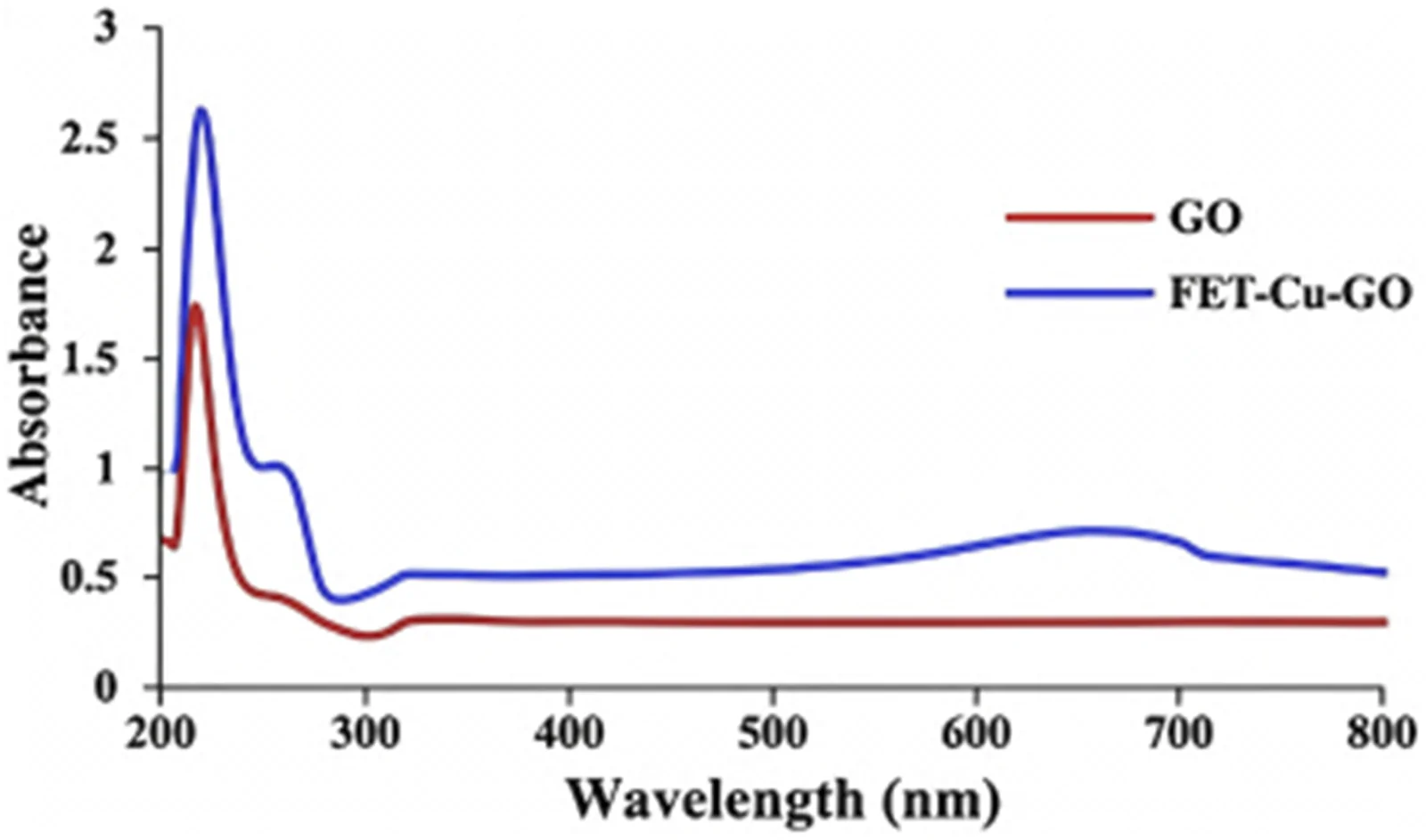
UV-vis spectra of pristine GO and the synthesized FET-Cu–GO-hybrid.

The FET-Cu–GO-hybrid spectrum exhibits a significant enhancement in absorbance across the entire ultraviolet-visible region, accompanied by the emergence of a broad, distinct absorption band centered around 600–700 nm. This low-energy feature is assigned to the d–d electronic transitions of the Cu(ii) center within the distorted square-planar or octahedral coordination sphere of the Schiff base ligand. Furthermore, the intensification of the ultraviolet bands and the emergence of a new shoulder near 350–400 nm are indicative of ligand-to-metal charge transfer (LMCT) processes between the imine nitrogen/phenolate oxygen and the copper center. The substantial overlap and synergistic enhancement of the absorption bands in the FET-Cu–GO hybrid, compared to the relatively featureless visible profile of pristine GO, provide compelling evidence for the chemical anchoring of the active metal complex. This optimized electronic structure not only confirms the hybrid formation but also suggests a highly photo-active or redox-responsive environment conducive to superior catalytic performance.^41^

#### 3.3.9. Optimization and kinetic modulation of the electrically assisted catalytic interface

To delineate the operational limits and intrinsic potential of the FET-Cu–GO-hybrid, we systematically explored the reaction manifold using the oxygenation of methyl phenyl sulfide as a probe transformation. The hybrid architecture demonstrated an extraordinary catalytic activity even at a minimal loading of 1 mg, underscoring a high density of highly accessible active sites. This efficiency was further amplified by the use of ethanol, which was found to facilitate the interfacial charge distribution between the catalyst and the reactants, thereby stabilizing the transition state. The strategic incorporation of 75 mmol of imidazole served as a critical coordinating axial ligand, modulating the electronic density of the copper centers to facilitate a more efficient oxygen-transfer cycle from the oxidant to the sulfide substrate.

The most profound kinetic acceleration was triggered by the imposition of an external electric field, revealing a critical electronic critical potential at 55 V. A progressive increase in product conversion was observed as the potential rose, with a sharp transition to quantitative yield (100%) specifically at 55 V, suggesting that this potential is the essential energetic requirement to polarize the substrate–catalyst transition state and overcome the potential-dependent activation barrier. Temporal analysis revealed a rapid ascent in yield, reaching its maximum at 78 seconds. This synergy between the electroactive copper centers and the conductive graphene oxide scaffold, acting as a high-flux electronic conduit, effectively transforms a traditionally sluggish process into an ultrafast catalytic event that significantly surpasses of conventional heterogeneous catalysis (Table 2).

**Table 2:** Influence of applied potential and reaction time on the electrocatalytic epoxidation of MePhS using FET-Cu–GO-hybrid

Voltage (V)	Time (s)	Conversion%
0	100	0
2	100	5
4	100	11
8	100	19
10	100	31
20	100	47
30	100	61
40	100	79
50	100	91
55	100	100
55	80	100
55	78	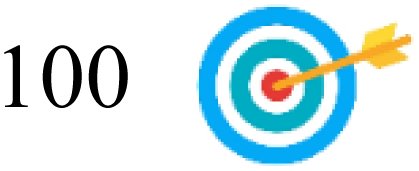
55	75	96

#### 3.3.10. Comparative catalytic performance and the impact of support interaction on activity

To delineate the necessity of the active metal centers and the pivotal role of the support, a series of comparative evaluations were performed. As evidenced by Fig. 11, both the blank control and pristine GO exhibited negligible conversion, confirming that the reaction is kinetically hindered without a transition-metal catalyst and that the GO framework itself is catalytically inactive. The introduction of the copper complex triggered a profound shift in performance; while the molecular CuL species demonstrated substantial activity (93% yield), its immobilization onto the graphene oxide sheets (FET-Cu–GO-hybrid) pushed the conversion to 100% and ensured superior structural stability. The nature of this interaction was further verified by examining the catalytic activity of a physical mixture of the complex and GO, which showed a marked decline in performance with a yield of only 73%. This deficiency is directly rooted in the absence of chemical bonding, as the GO sheets act as steric obstructions that shield the active metal centers, thereby restricting substrate accessibility and impeding efficient electron transport. In contrast, the hybrid system eliminates these limitations through covalent integration, leveraging the intrinsic conductivity of GO to maximize the exposure of active sites. The superior efficiency of the FET-Cu–GO-hybrid is the result of the synergistic interplay between the high-surface-area GO matrix and the coordinated Cu(ii) centers, which precludes the aggregation of active species and merges molecular reactivity with macroscopic robustness. This transition from a homogeneous state to a heterogeneous platform proves the criticality of the GO support in establishing a recyclable catalytic system.

**Fig. 11:**
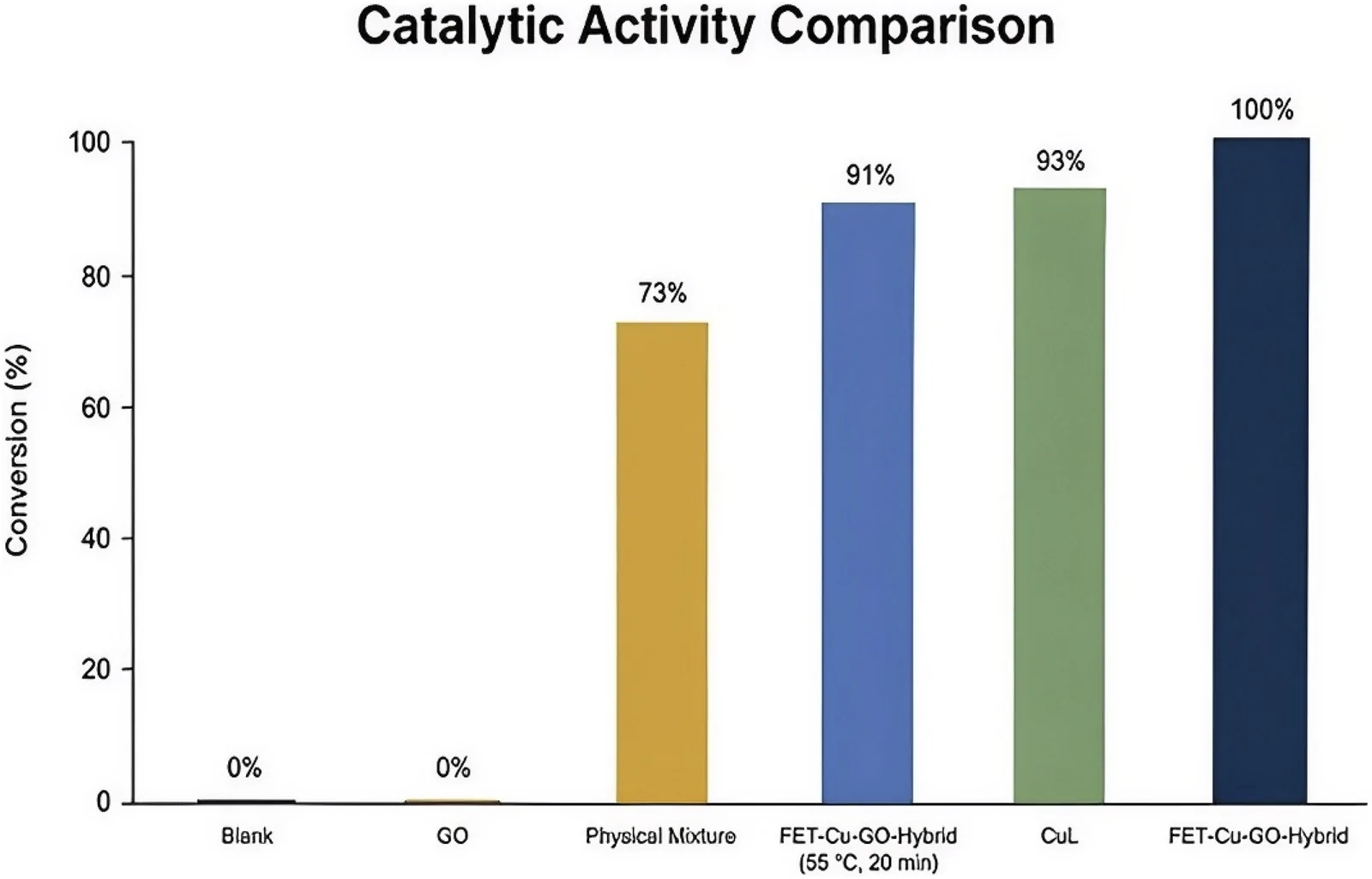
Evaluation of the catalytic performance of the GO support, homogeneous CuL, and heterogeneous FET-Cu–GO-hybrid in the oxidation of methyl phenyl sulfide. Reaction conditions: catalyst (1 mmol), methyl phenyl sulfide (350 mmol), 30% H_2_O_2_ (1600 mmol), imidazole (75 mmol), ethanol (1 mL), room temperature, and 78 s duration under an applied potential of 55 V.

#### 3.3.11. Operational robustness and recyclability: assessing the structural integrity of FET-Cu–GO-hybrid

The stability and reusability of the FET-Cu–GO-hybrid were evaluated to assess its potential for practical applications. Under optimized conditions (78 s, 55 V), the nanocomposite exhibited consistent catalytic activity over six consecutive cycles. To ensure the integrity of the catalyst, the material was thoroughly washed with absolute ethanol the solvent used for the homogeneous CuL complex after each run to remove any potentially adsorbed species. The maintenance of high yields despite this rigorous washing suggests that the active sites are firmly anchored to the support. This was further confirmed by Atomic Absorption Spectroscopy (AAS) analysis of both the filtrate and the recovered catalyst after the sixth cycle, which revealed no detectable metal leaching and stable copper content, respectively. Such stability is attributed to the strong coordination between the copper species and the functionalized GO matrix, which prevents detachment even under the influence of an external electric field. Furthermore, the stable current response observed in chronoamperometric measurements and the field-enhanced kinetics which fundamentally require the immobilization of active species on a conductive surface provide additional evidence that the FET-Cu–GO-hybrid functions as a heterogeneous catalyst. These findings highlight the material's suitability for sustainable oxidation, offering an optimal balance between catalytic efficiency and long-term durability.^42^

#### 3.3.12. Electrochemical behavior and electrocatalytic activity of FET-Cu–GO-hybrid

The electrocatalytic behavior of the FET-Cu–GO-hybrid was investigated to evaluate the charge transfer kinetics and the influence of the conductive GO scaffold on the overall reaction rate. The cyclic voltammetry (CV) profiles reveal a significant shift in the oxidation potentials toward lower values when compared to the blank system. This observed reduction in overpotential suggests that the hybrid catalyst effectively lowers the activation energy barrier required for electron transfer from the substrate to the electrode surface, thereby facilitating a more rapid oxidation process. Furthermore, the well-defined reduction peaks observed in the cathodic region indicate the efficient regeneration of the active Cu(ii) centers. This reversible behavior is critical for maintaining a continuous catalytic cycle, ensuring that the active sites are restored without compromising the structural integrity of the complex. To further understand the nature of the electron transfer, the relationship between the peak current (*I*_p_) and the scan rate (*v*) was analyzed. The linear correlation observed indicates a surface-controlled electrochemical process. In this mechanism, the functionalized graphene oxide acts as an effective reservoir that increases the local concentration of reactants at the electrode interface, which in turn optimizes the collision frequency and enhances the overall kinetic rate. The long-term kinetic stability was further validated using chronoamperometry. While the blank system exhibited a rapid decay in current, the FET-Cu–GO-hybrid maintained a stable current plateau over the analyzed period. This result confirms that the GO framework facilitates efficient charge transport and prevents the accumulation of inactive species at the interface, ensuring a steady-state reaction rate throughout the process (Fig. 12). The structural stability of the catalyst was verified by atomic absorption spectroscopy (AAS), which showed no detectable metal leaching after six consecutive catalytic cycles. This high level of stability is attributed to the strong coordination bonds between the copper centers and the functionalized oxygen groups of the GO matrix. Such robust anchoring prevents the detachment of the metal complex, even under the influence of an external electric field. The combination of reduced oxidation potentials, optimized electron flux, and structural robustness explains Cu–GO hybrid, positioning it as a high-performance interface for the rapid and selective oxygenation of organic sulfides.^43,44^

**Fig. 12:**
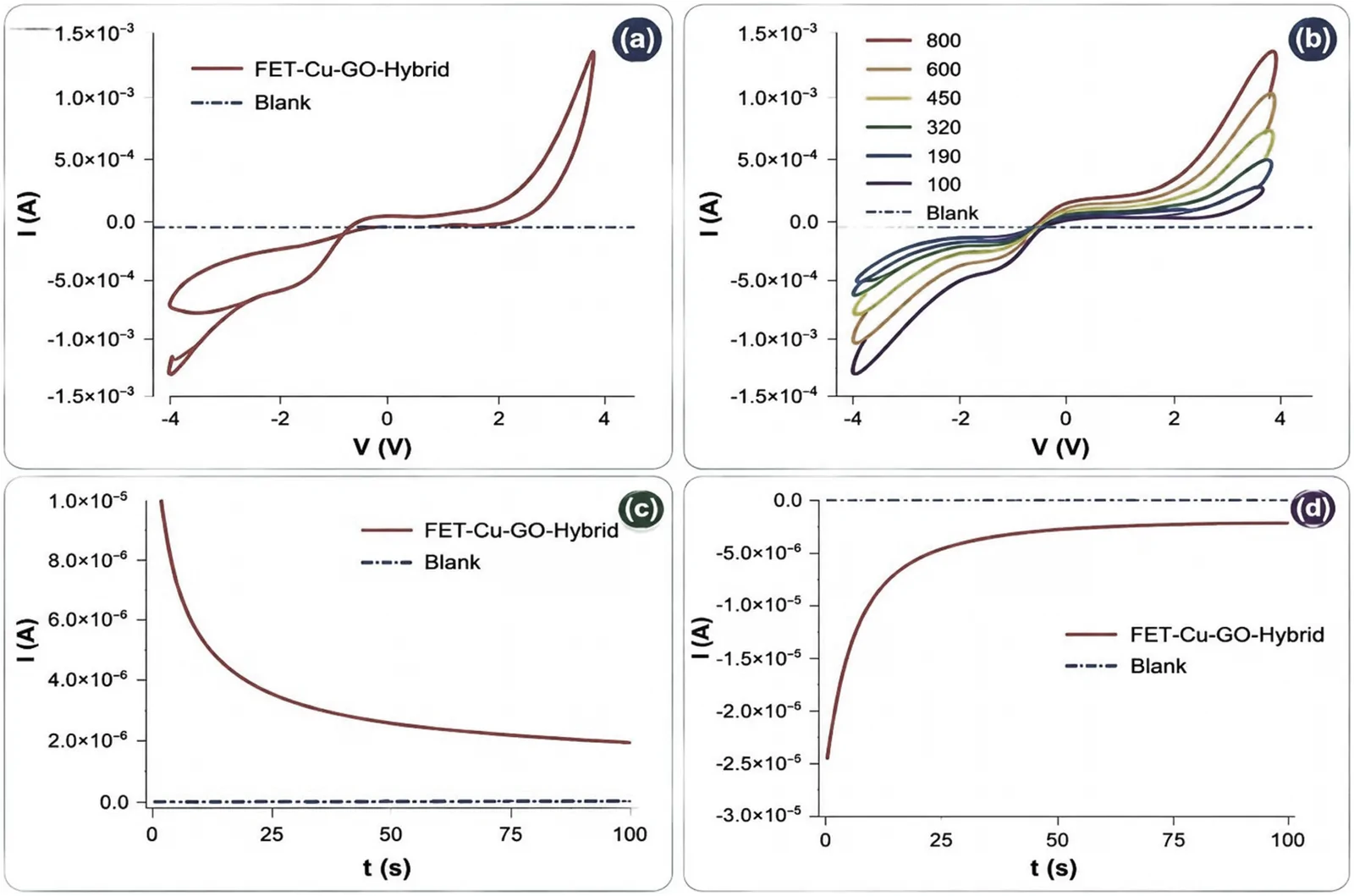
Cyclic voltammograms (CV) of FET-Cu–GO-hybrid and the blank (control) reaction for the oxidation of methyl phenyl sulfide at a scan rate of 100 mV s^−1^. (b) Cyclic voltammograms of FET-Cu–GO-hybrid at scan rates of 100, 200, 400, 600, 800, and 800 mV. Chronoamperometric profiles of FET-Cu–GO-hybrid, and the blank electrode at (c) the oxidation potential and (d) the reduction potential. Reaction conditions: catalyst (1 mmol), methyl phenyl sulfide (350 mmol), 30% H_2_O_2_ (1600 mmol), imidazole (75 mmol), ethanol (1 mL), room temperature, and 78 s duration under an applied potential of 55 V.

#### 3.3.13. Exploring the catalytic proficiency and chemoselectivity for a wide range of sulfide derivatives

The synthetic utility of the FET-Cu–GO-hybrid was rigorously evaluated across a diverse library of sulfide substrates to assess its catalytic robustness and chemoselectivity. As summarized in the results the hybrid catalyst exhibited exceptional performance yielding near quantitative conversion and absolute selectivity toward the corresponding sulfoxides. A pivotal observation is the exceptional chemoselectivity demonstrated by the system. For across the entire substrate scope tested the exclusive formation of sulfoxides regardless of the conversion rate. This indicates a total suppression of over oxidation to sulfones across the entire substrate scope. This precise control over the oxidation state is attributed to the finely tuned electronic environment of the Cu centers anchored on the GO scaffold which facilitates the immediate release of the sulfoxide product before any further oxidation can occur.

Furthermore, the catalyst displayed remarkable tolerance toward various electronic and steric profiles of the sulfide R groups. While the conversion rates varied slightly from 83% to 100% depending on the substrate the selectivity remained consistently at 100%. This proves that the catalyst maintains its intrinsic chemoselectivity irrespective of the substrates steric bulk or electronic nature. Such a high degree of precision combined with the synergistic effect of the conductive GO framework positions the FET-Cu–GO-hybrid as a highly efficient and sustainable platform for selective organic transformations (Table 3).

**Table 3:** Evaluation of the substrate scope and chemoselectivity of the FET-Cu–GO-hybrid in the electro-assisted oxidation of various organic sulfides to sulfoxides^a^

Sulfides	Conversion%	Sulfoxide product%
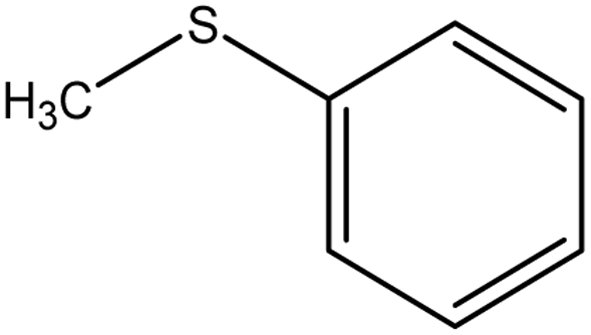	100	100
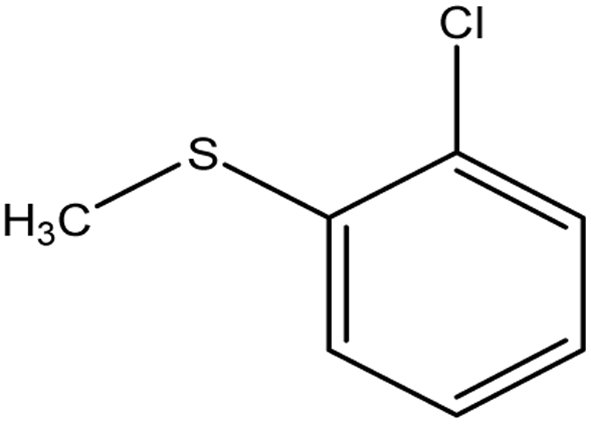	94	100
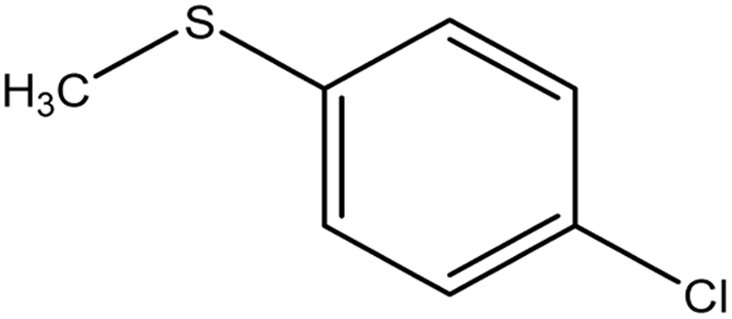	100	100
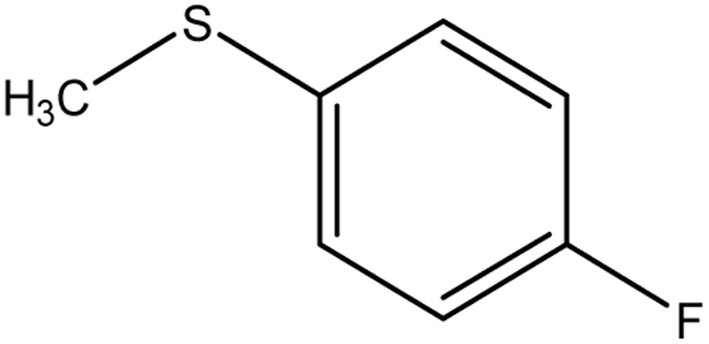	89	100
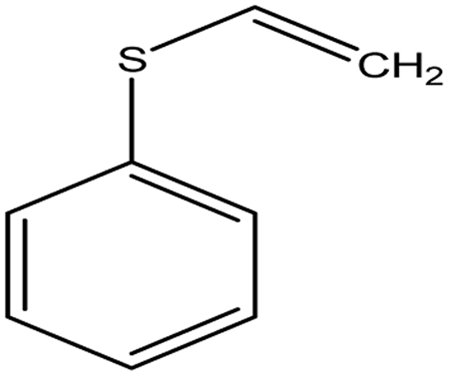	100	100
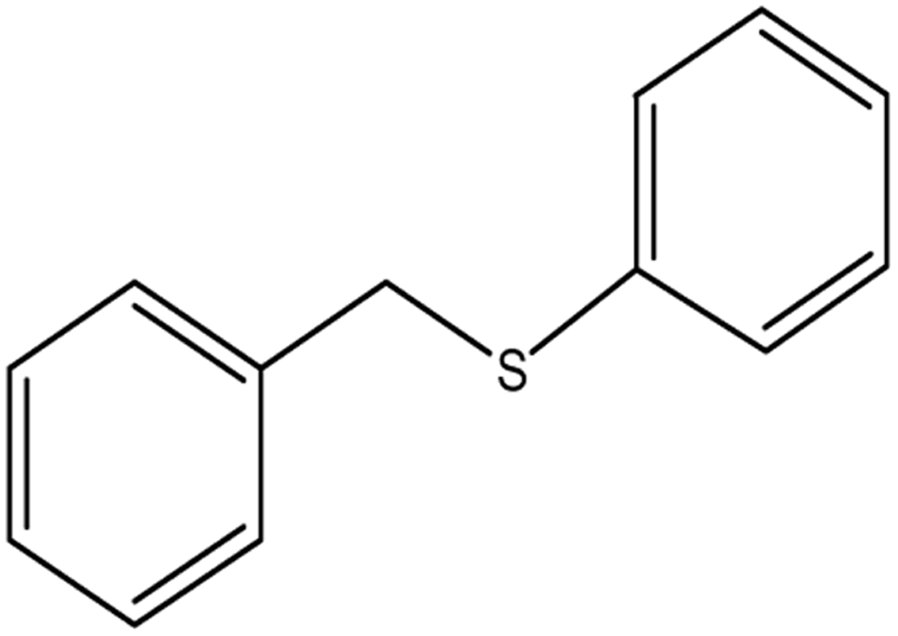	91	100
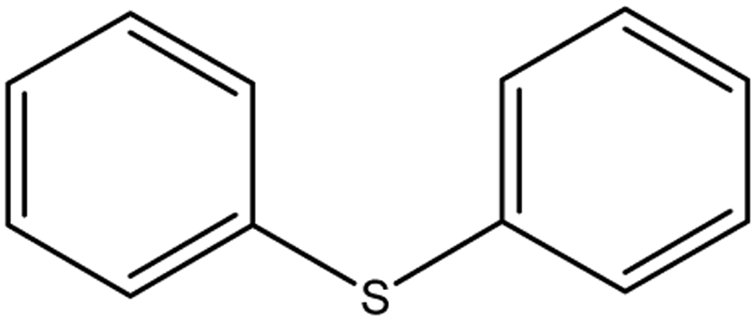	83	100
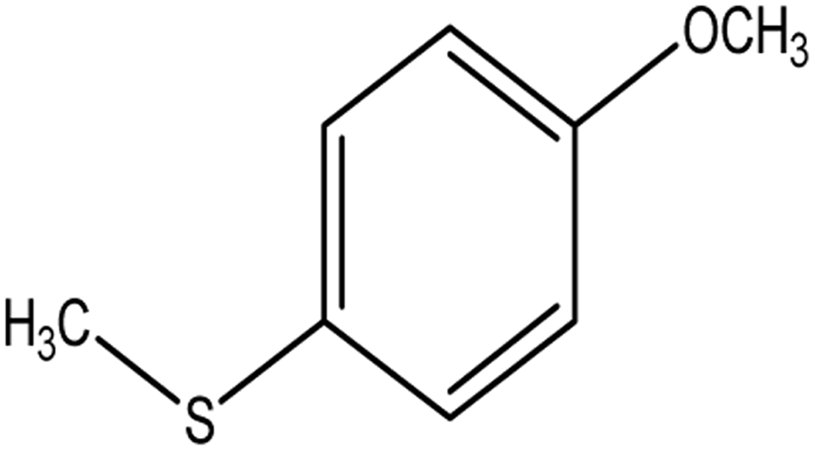	100	100
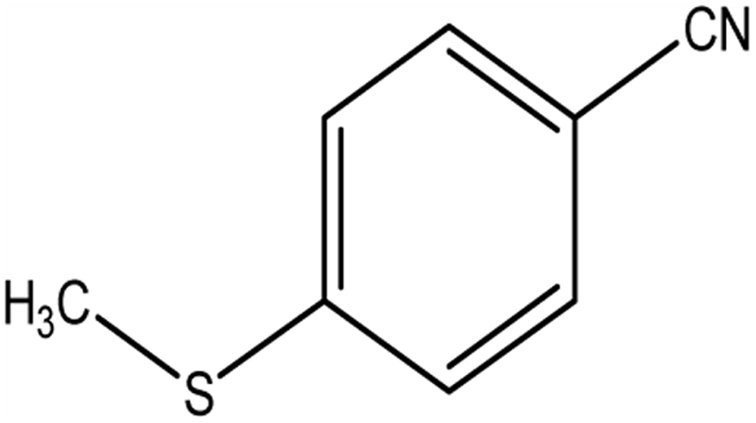	98	100
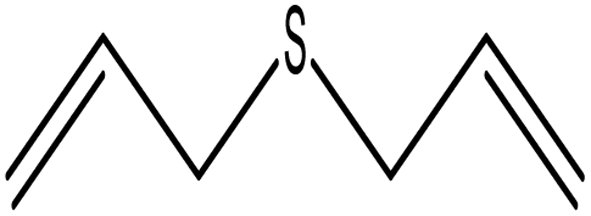	100	100
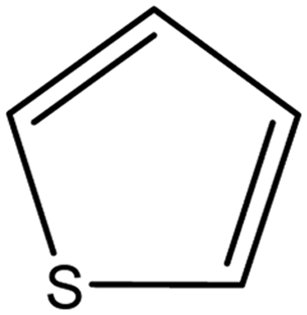	100	100

a Reaction conditions: catalyst (1 mmol), methyl phenyl sulfide (320 mmol), 30% H_2_O_2_ (1600 mmol), imidazole (75 mmol), ethanol (1 mL), room temperature, and 90 s duration under an applied potential of 55 V.

#### 3.3.14. Mechanistic insights and synergistic effects

The proposed catalytic cycle illustrates the synergy between the electrochemical potential and the redox flexibility of the copper centers anchored on graphene oxide (GO). The process begins at the Cu(ii) resting state, where the application of a cathodic potential triggers the reduction of the metal center to Cu(i). This active species subsequently activates H_2_O_2_ to generate a highly reactive Cu(ii)–OOH intermediate. The efficiency of this transition is facilitated by the sp^2^-hybridized network of GO, which serves as an electronic conduit, enhancing electron transfer kinetics and reducing the overpotential required for the Cu(ii)/Cu(i) redox shuttle. Additionally, the high surface area of GO prevents the aggregation of active sites, ensuring a high distribution of accessible Cu centers. The stability of the system over multiple cycles is attributed to the robust anchoring of the complex to the GO support *via* stable ester linkages, which effectively minimizes catalyst leaching. Mechanistically, the formation of the Cu(ii)–OOH species lowers the activation energy (*E*_a_) for oxygen atom transfer (OAT) to the sulfide substrate through a concerted transition state, streamlining the reaction pathway. Finally, the catalyst is regenerated to its initial Cu(ii) state *via* an anodic oxidation process, ensuring long-term stability under potential-driven control (Fig. 13).

**Fig. 13:**
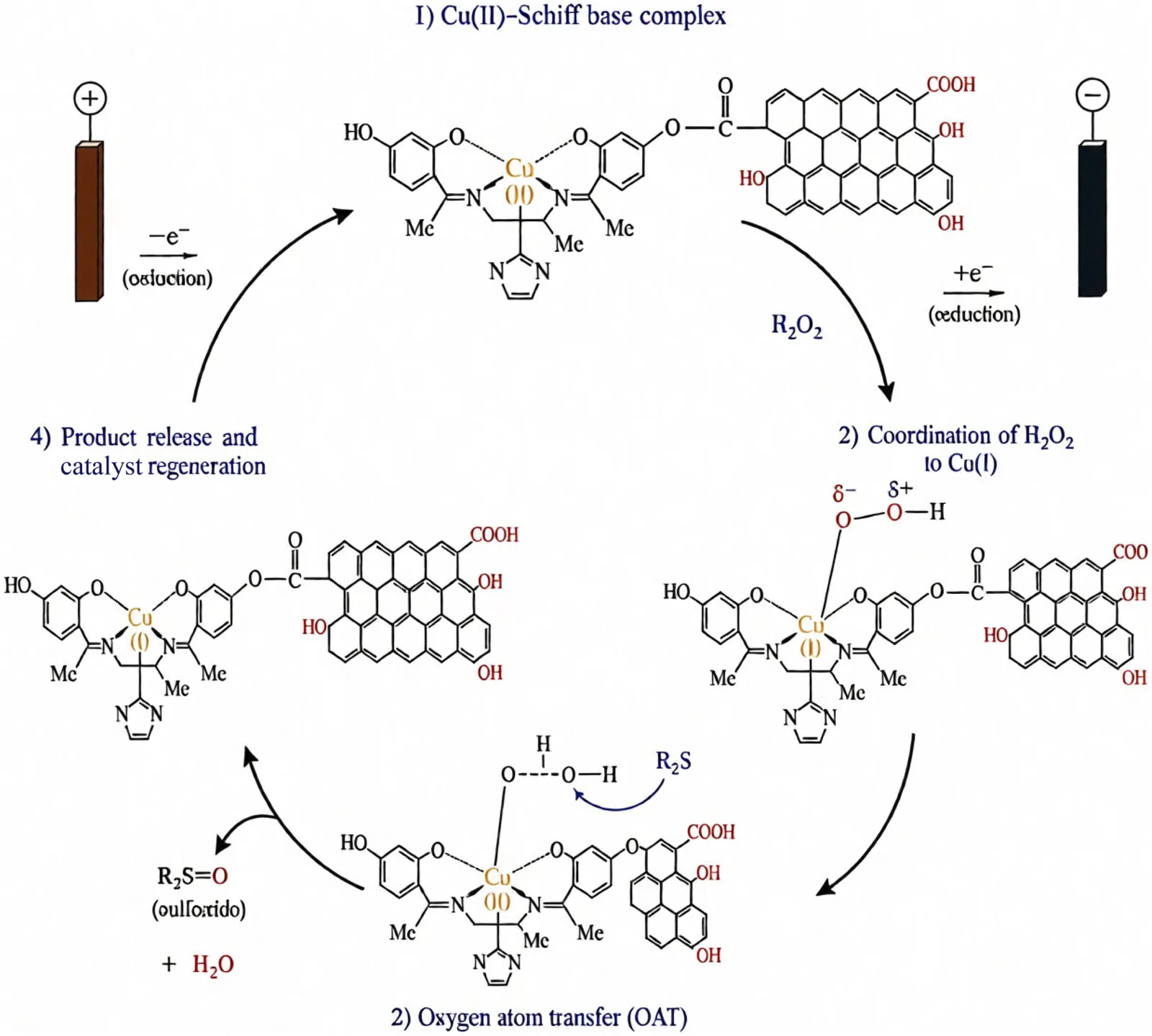
Schematic representation of the proposed electrochemical catalytic cycle for the oxidation of sulfide.

The observed increase in reaction rate under an electric field is attributed to the modulation of electronic energy levels and optimized charge kinetics.^45^ In the absence of an external field, the reaction is hindered by a high kinetic barrier arising from energy mismatch and poor spatial overlap between the Cu d-orbitals and the H_2_O_2_ LUMO orbitals. The applied electric field induces a shift in these electronic levels (consistent with the Stark effect) which lowers the H_2_O_2_ LUMO and aligns it with the d-band center of the Cu active sites.^46^ This alignment enhances orbital overlap, thereby reducing the activation energy for the oxidative addition of H_2_O_2_ and the subsequent formation of the Cu-peroxo species.^47^ Furthermore, the electric field utilizes a donor–acceptor mechanism to modulate the oxidation states of the reactants and the catalyst, allowing the system to optimize adsorption–desorption energetics beyond the traditional Sabatier maximum.^48^ In this architecture, the conductive GO scaffold acts as an electron reservoir that ensures rapid charge transport and minimizes the overpotential for the Cu(i)/Cu(ii) transition, facilitating efficient inner-sphere electron transfer from the sulfide substrate. Consequently, the reduction in transition state energy and the improved electronic coupling *via* the GO framework lead to a significant increase in the overall reaction rate.

## 4 Conclusions

This study demonstrates the strategic development of the FET-Cu–GO-hybrid catalyst, specifically engineered to address the persistent challenges of stability and activity in the electro-oxidative synthesis of sulfoxides. The heterogeneous catalyst was meticulously synthesized and its structure rigorously characterized; the crystal structure of the copper(ii)-Schiff base complex was precisely determined, confirming its successful grafting onto graphene oxide *via* stable covalent linkages. This structural integrity effectively eliminated metal leaching a critical drawback in conventional copper systems. A pivotal advancement of this work is the integration of electrochemical potential tuning, allowing for the precise modulation of the active sites' electronic states. This synergy between the conductive carbon framework and voltage control resulted in an unprecedented acceleration of the reaction, achieving complete conversion in only 78 seconds. Operating in a sustainable ethanol medium, the process strictly adheres to the principles of green chemistry by eliminating toxic solvents and minimizing waste, while the exceptional stability (TON of 46 000) and excellent recyclability underscore the catalyst's robustness. Ultimately, this work provides a scalable and sustainable framework for the design of high-performance hybrid catalysts, bridging the gap between rapid kinetic performance and long-term catalytic durability in organic transformations.

## Author contributions

E. Khodaei: conceptualization, methodology, software, validation, formal analysis, investigation, resources, data curation, writing – original draft, writing – review and editing, visualization, supervision, project administration, funding acquisition. A. Abbasi: writing – review and editing.

## Conflicts of interest

The authors declare no conflict of interest.

## Supplementary Material

RA-OLF-D6RA06156G-s001

RA-OLF-D6RA06156G-s002

## Data Availability

Data will be available on reasonable request to corresponding authors. CCDC 1537879 contains the supplementary crystallographic data for this paper.^49^ Supplementary information (SI) is available. See DOI: https://doi.org/10.1039/d6ra06156g.

## References

[cit1] Fan Q., Liu D., Liao Z., Wu D., Xie Z., Zhu H., Le Z. (2025). J. Catal..

[cit2] Addadi K., Belboukhari N., Sekkoum K. (2025). Mediterranean Journal of Medical Research.

[cit3] Hu Y., Li N., Wang M., Qiao Z., Gu D., Zhu L., Yuan D., Wang B. (2025). Catalysts.

[cit4] Ajiboye T. O., Mafolasire A. A., Aruada C., Ogunbiyi O. D., Dhibar S., Nthunya L. N. (2025). Discover Appl. Sci..

[cit5] Gulliani S., Volpe M., Messineo A., Volpe R. (2023). RSC Sustainability.

[cit6] Huang H., Xie X., Xiao F., Liu B., Zhang T., Feng F., Lan B., Zhang C. (2024). Environ. Sci. Technol..

[cit7] Gupta S. N. (2025). ChemCatChem.

[cit8] Sarfraz H., Shabanian S., Shabbir S., Khan A. U., Khan Y. (2026). Pure Appl. Chem..

[cit9] Li S., Xia S., Ma X., Du L., Li Y., Tang Z., Wu Z., Zhang W. (2026). Carbon Neutralization.

[cit10] Wayo D. D. K., Goliatt L., Ganji M. D. (2025). Rev. Chem. Eng..

[cit11] Nguyen H. T., Nguyen T. T., Doan V. T. C., Nguyen T. H., Tran M. H. (2025). RSC Adv..

[cit12] Wang D., Wang Z., Xiong J., Yu Z., Lu X. (2025). ChemCatChem.

[cit13] Metla S. B. S., Rouhani M., Hobley J., Ou H. H., Jeng Y. R. (2026). Applied Surface Science Advances.

[cit14] Aliyeva N., Baskan-Bayrak H., Aleinawi M. H., Erdem E., Paterakis G., Galiotis C., Al Hajri E., Samad Y. A., Okan B. S. (2026). J. Energy Storage.

[cit15] Pathan I. R., Patel M. K. (2023). Inorg. Chem. Commun..

[cit16] Cristóvão B., Osypiuk D., Bartyzel A. (2022). Materials.

[cit17] Cao K., Tian Z., Zhang X., Wang Y., Zhu Q. (2023). Sci. Rep..

[cit18] Taghiyeva N., Hasanova U., Millet M., Gardiennet C., Gakhramanova Z., Mirzayev M. H., Gahramanli L. (2024). Materials.

[cit19] Momeni S., Ghorbani-Vaghei R. (2025). Sci. Rep..

[cit20] Zhang K., Pang T., Song J., Mansoor M., Zhong J., Yan T., Xie M., Cheng D., Zhang D. (2025). ACS Appl. Mater. Interfaces.

[cit21] Abhishek Anil K., Kantesaria R., Panda H. S. (2026). Discover Chemistry.

[cit22] Sheikh A. H., Farhana A., Nethaji K., Baildya N., Madhu N. R., Choudhury N. A., Shanmugam P., Pathak S., Banerjee A., Adhikari S. (2025). J. Coord. Chem..

[cit23] Fadhil Z. S., Hassan Q. M. A., Hussein K. A., Sultan H. A., Al Shawi J. M. S., Emshary C. A. (2024). Phys. Scr..

[cit24] Malav R., Ray S. (2025). RSC Adv..

[cit25] Elantabli F. M., Mohamed R. G., El-Medani S. M., Haukka M., Ramadan R. M., Afifi M. A. (2024). J. Mol. Struct..

[cit26] Ali M., Ullah H., Khan R., Islam N. U., Khan E., Ahmad M., Ullah A., Begum A., Ali G., Xie Y. (2026). RSC Adv..

[cit27] Inac H., Ashfaq M., Dege N., Feizi-Dehnayebi M., Munawar K. S., Yağcı N. K., Çınar E. P., Tahir M. N. (2024). J. Mol. Struct..

[cit28] Mandal S., Sarkar M., Denrah S., Bagchi A., Biswas A., Cordes D. B., Slawin A. M. Z., Saha N. C. (2023). J. Mol. Struct..

[cit29] Fathalla E. M., Morsy Abu-Youssef A. M., Sharaf M. M., El-Faham A., Barakat A., Haukka M., Soliman S. M. (2023). Inorganics.

[cit30] Ebrahimipour S. Y., Khajoee Nejad F., Khosravan M., Castro J., White J. M., Mohammadi P., Mohamadi-Nejad G., Mohamadi M. (2025). J. Mol. Struct..

[cit31] Tafazzoli A., Keypour H., Moazzami Farida S. H., Ahmadvand Z., Gable R. W. (2023). J. Mol. Struct..

[cit32] Sundaram G. A., Kumaravelu S., Tseng W. L., Pham P. V., Kumar A. S. K., Parimelazhagan V. (2024). Materials.

[cit33] Zhang Z., Wang X., Tian H., Jiao H., Tian N., Bian L., Liu Y., Wang Z. L. (2024). J. Colloid Interface Sci..

[cit34] Brusko V., Khannanov A., Rakhmatullin A., Dimiev A. M. (2024). Carbon.

[cit35] Abdel-Rahman L. H., Mohammed K. M. H., Ahmed E. A. H., Abdelghani A. A., Nafady A. (2025). J. Inorg. Organomet. Polym. Mater..

[cit36] Tahmasbi B., Darabi M., Moradi P., Tyula Y. A., Nikoorazm M. (2024). Polyhedron.

[cit37] Patel U. K., Agarwal A. (2025). Diamond Relat. Mater..

[cit38] Babapoor A., Asgharzadeh R., Pourshahi M. (2024). Chem. Afr..

[cit39] Alghamdi A. M., Ali A. (2025). RSC Adv..

[cit40] Rashid M., Kouser R., Arjmand F., Tabassum S. (2023). Opt. Mater..

[cit41] Wu B., Hong M., Wu Q., Li X., Zhao Y., Wang S., Wang Z. (2025). Environ. Res..

[cit42] Moeketse T. N., Baker P. G., Makgwane P. R. (2025). Electrochim. Acta.

[cit43] Alshahrani S. M., Alkhammash A., Alamoudi Z. A. (2025). Diamond Relat. Mater..

[cit44] Kaplunenko V. and Kosinov M., Electric Field-Induced Catalysis. Laws of Field Catalysis, 2025

[cit45] Pan Y., Wang X., Zhang W., Tang L., Mu Z., Liu C., Tian B. (2022). Nat. Commun..

[cit46] Fortunato G. V., Pizzutilo E., Katsounaros I., Göhl D., Lewis R. J., Mayrhofer K. J. J., Hutchings G. J., Freakley S. J., Ledendecker M. (2022). Nat. Commun..

[cit47] Léonard N. G., Dhaoui R., Chantarojsiri T., Yang J. Y. (2021). ACS Catal..

[cit48] Kosinov M. V. and Kaplunenko V. G., US Pat., UA150017, 2021

[cit49] CCDC 1537879: Experimental Crystal Structure Determination, 2026, 10.5517/ccdc.csd.cclnm901

