# Supplementary material for: Highly efficient electro-oxidative synthesis of sulfoxides: synergistic effect of an optimized copper(ii) complex and covalently functionalized graphene oxide nanohybrid
Source: RSC Adv. 2026 Sep 29;16(45):58312–28. doi: 10.1039/d6ra06156g (PMC13621346; doi:10.1039/d6ra06156g)
Supplement: Supplementary file 1 [file RA-016-D6RA06156G-s001.pdf]

Supplementary Information (SI) for RSC Advances.
This journal is © The Royal Society of Chemistry 2026

Supporting Information

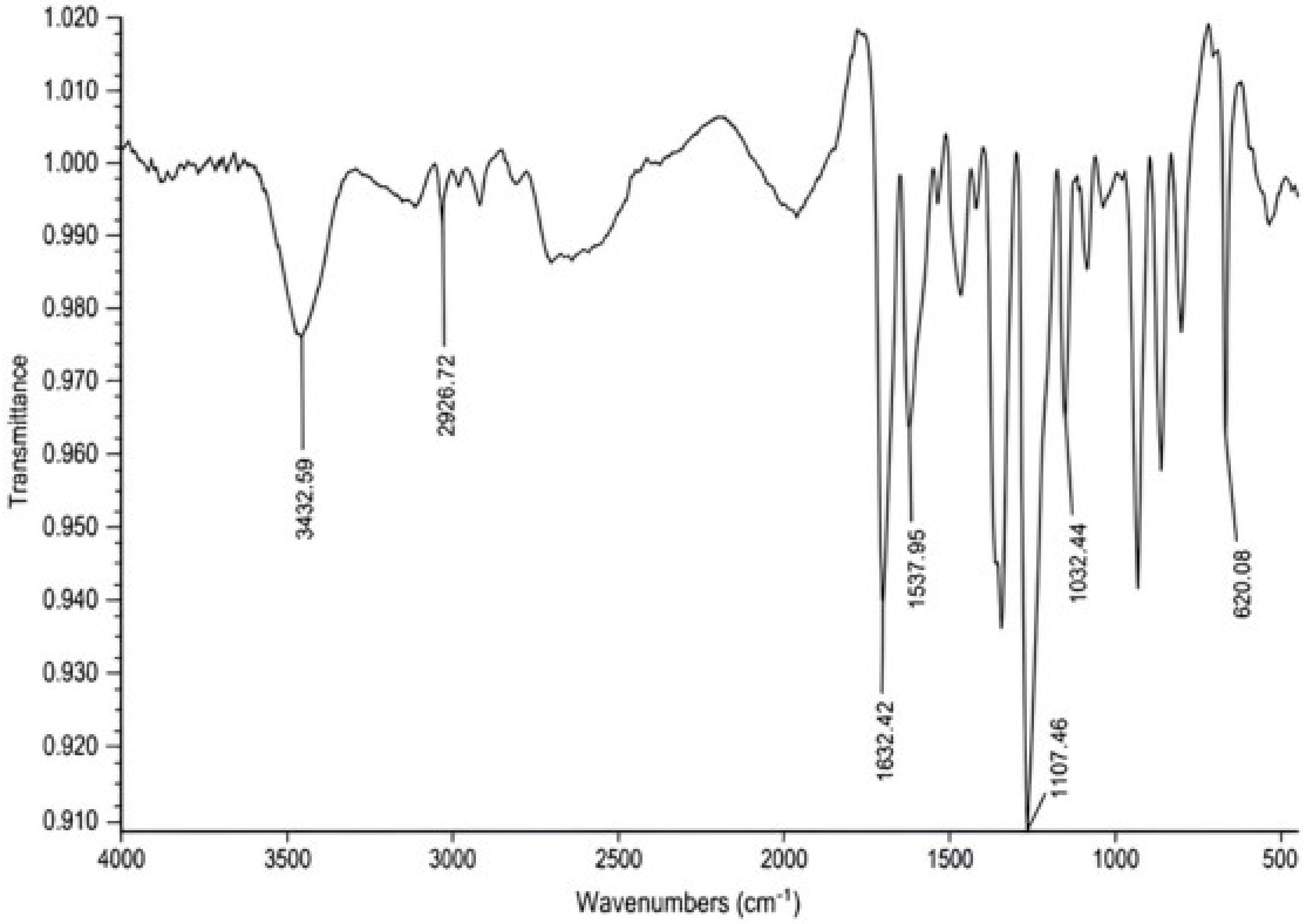

Figure S1. IR spectrum of the synthesized Schiff base ligand.

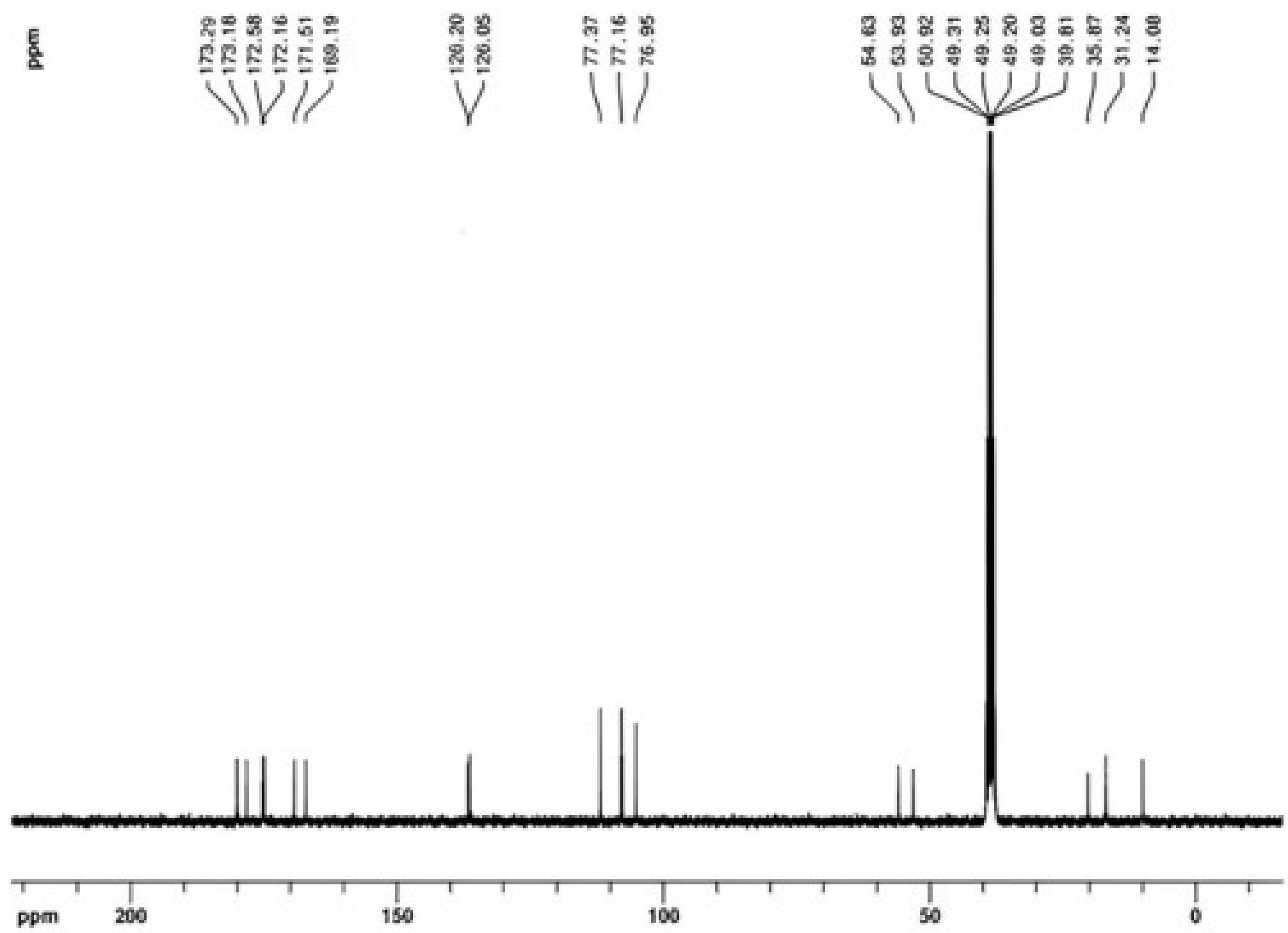

Figure S2. ^{1}CNMR spectrum (400 MHz, DMSO-d_6) of the synthesized Schiff-base ligand (H_2L)

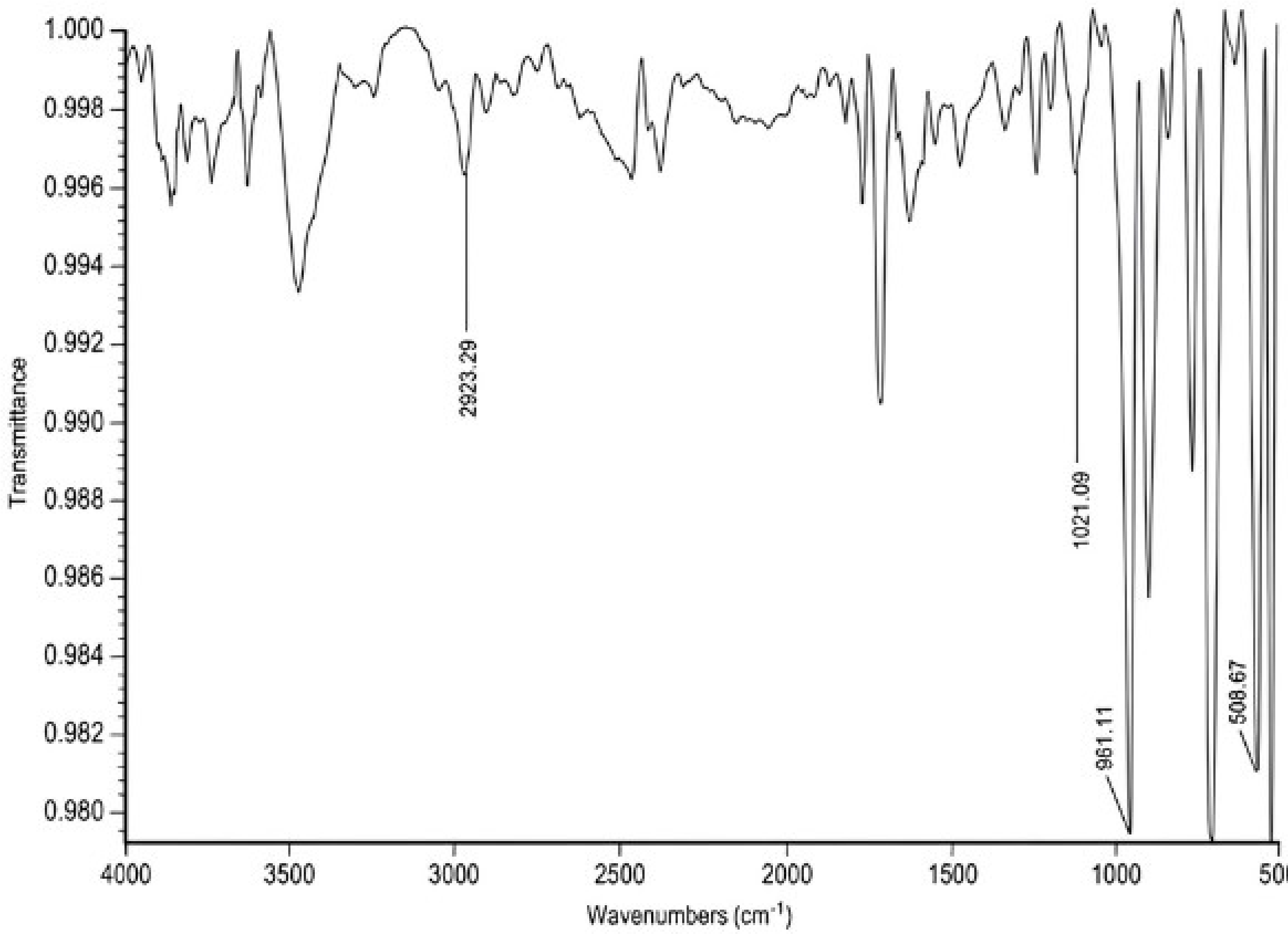

Figure S3. FT-IR spectra of the CuL complex

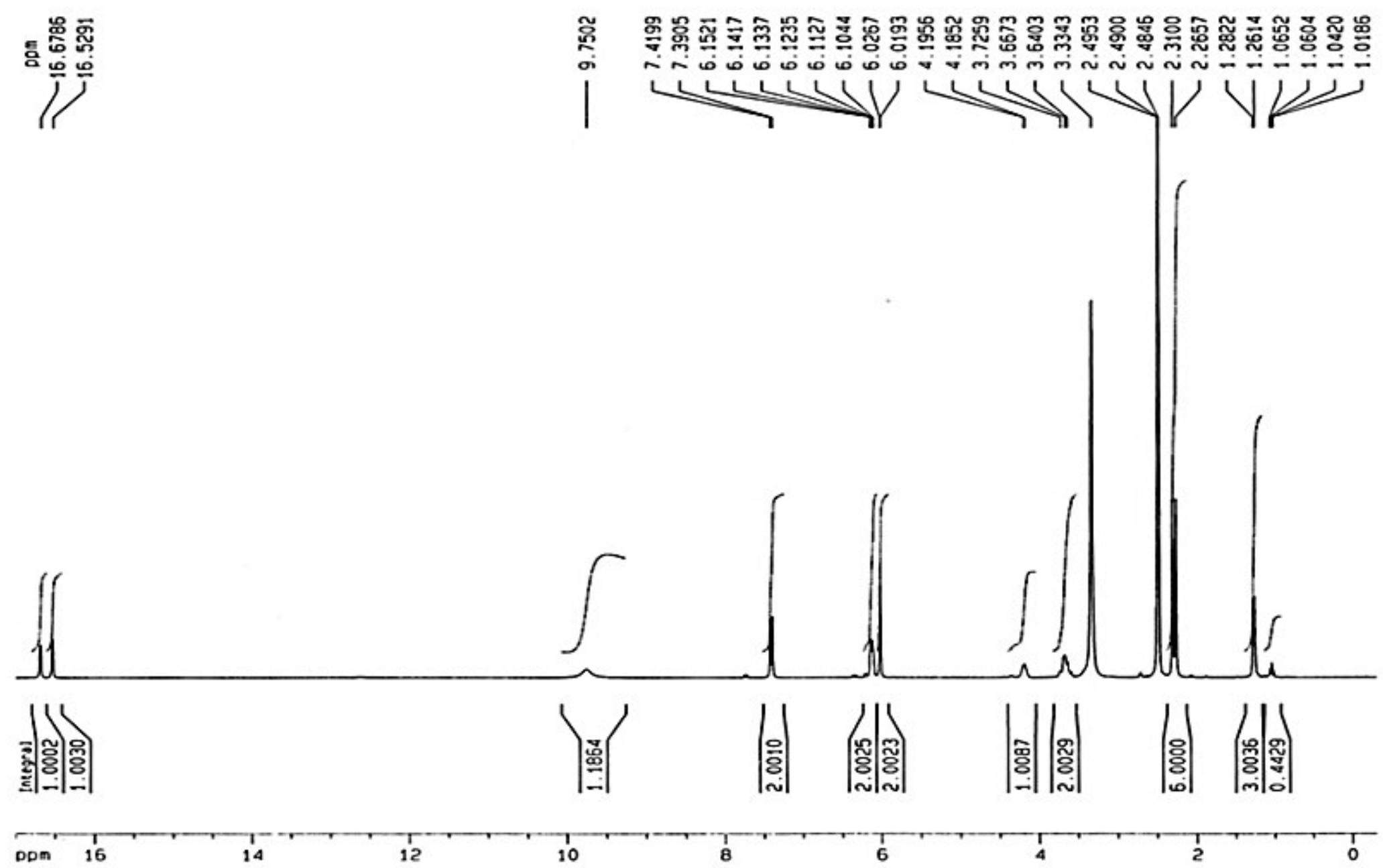

Figure S4. ^{1}HNMR spectrum (400 MHz, DMSO-d6) of the synthesized Schiff-base ligand (H_2L)

Table S1. Physicochemical characteristics, colorimetric observations, and thermal stability profiles of the synthesized Schiff-base Ligand (H_2L)

$C_{19}H_{22}N_2O_4$	**Conversion %**	**Color**	**Decomp. Temp.**
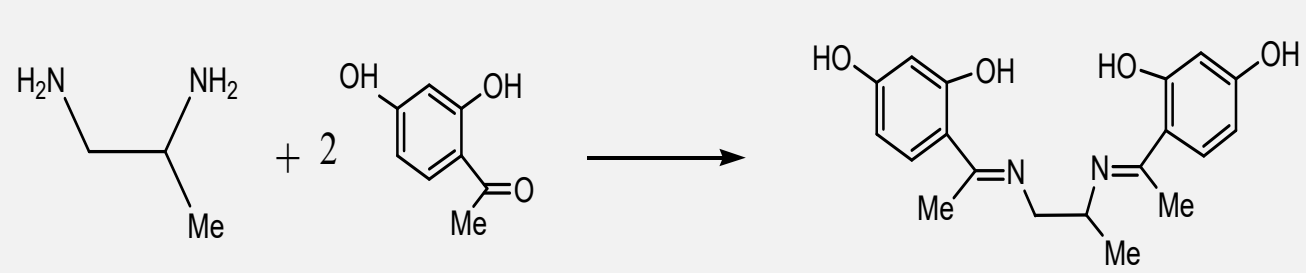	92	Yellow	**180 °C**

Table S2. Physicochemical characteristics, colorimetric observations, and thermal stability profiles of the synthesized Schiff-base Ligand (H_2L)

$C_{19}H_{22}N_2O_4$	**Conversion %**	**Color**	**Decomp. Temp.)**
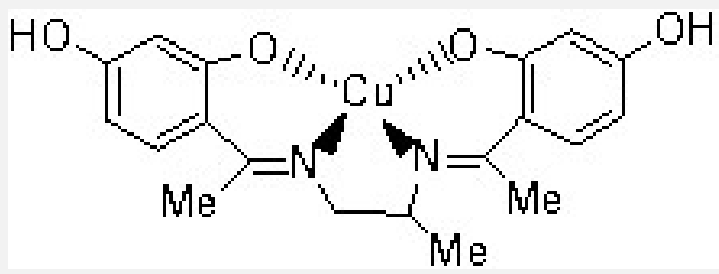	94%	Dark Brown	**265 °C**

Table S3. Electronic absorption data for the ligand H_2L

**Compound**	**λ_{max} (nm) (ε, mol^{-1}dm^{3}cm^{-1})**	**Assignment**
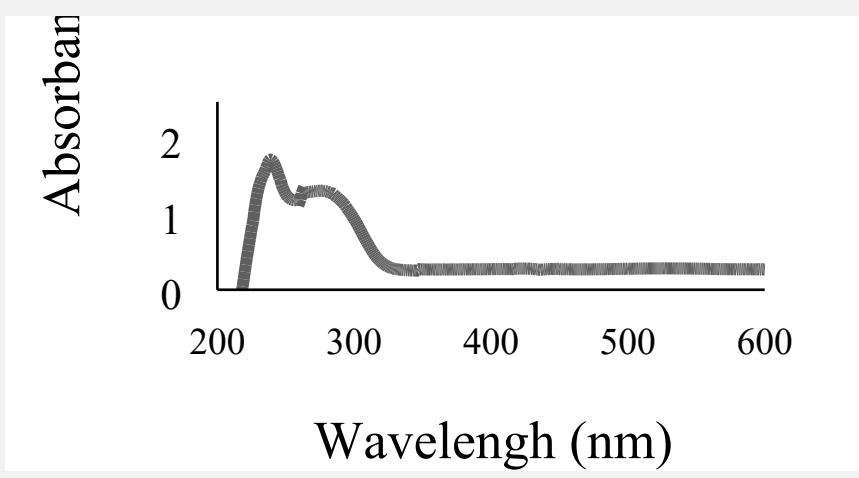	280<br>300	**π→π***<br>**n→π***
